# Phosphodiesterase and psychiatric disorders: a two-sample Mendelian randomization study

**DOI:** 10.1186/s12967-023-04368-0

**Published:** 2023-08-21

**Authors:** Miaomiao Jiang, Weiheng Yan, Yuyanan Zhang, Zhe Lu, Tianlan Lu, Dai Zhang, Jun Li, Lifang Wang

**Affiliations:** 1 National Clinical Research Center for Mental Disorders (Peking University Sixth Hospital), NHC Key Laboratory of Mental Health (Peking University), Peking University Sixth Hospital, Peking University Institute of Mental Health, Beijing, China; 2 Children’s Hospital Capital Institute of Pediatrics, Chinese Academy of Medical Sciences & Peking Union Medical College, Beijing, China; 3 Guangdong Key Laboratory of Mental Health and Cognitive Science, Institute for Brain Research and Rehabilitation (IBRR), South China Normal University, Guangzhou, China

**Keywords:** Phosphodiesterase, Psychiatric disorders, cAMP, cGMP, Mendelian randomization study

## Abstract

**Background:**

Phosphodiesterases (PDEs) have been associated with psychiatric disorders in observational studies; however, the causality of associations remains unestablished.

**Methods:**

Specifically, cyclic nucleotide PDEs were collected from genome-wide association studies (GWASs), including PDEs obtained by hydrolyzing both cyclic adenosine monophosphate (cAMP) and cyclic guanosine monophosphate (cGMP) (PDE1A, PDE2A, and PDE3A), specific to cGMP (PDE5A, PDE6D, and PDE9A) and cAMP (PDE4D and PDE7A). We performed a bidirectional two-sample Mendelian randomization (MR) analysis to investigate the relationship between PDEs and nine psychiatric disorders. The inverse-variance-weighted (IVW) method, MR-Egger, and weighted median were used to estimate causal effects. The Cochran’s Q test, MR-Egger intercept test, MR Steiger test, leave-one-out analyses, funnel plot, and MR pleiotropy residual sum and outlier (MR-PRESSO) were used for sensitivity analyses.

**Results:**

The PDEs specific to cAMP were associated with higher-odds psychiatric disorders. For example, PDE4D and schizophrenia (SCZ) (odds ratios (OR) = 1.0531, *P*_IVW_ = 0.0414), as well as major depressive disorder (MDD) (OR = 1.0329, *P*_IVW_ = 0.0011). Similarly, PDE7A was associated with higher odds of attention-deficit/hyperactivity disorder (ADHD) (OR = 1.0861, *P*_IVW_ = 0.0038). Exploring specific PDE subtypes and increase intracellular cAMP levels can inform the development of targeted interventions. We also observed PDEs (which hydrolyzes both cAMP and cGMP) was associated with psychiatric disorders [OR of PDE1A was 1.0836 for autism spectrum disorder; OR of PDE2A was 0.8968 for Tourette syndrome (TS) and 0.9449 for SCZ; and OR of PDE3A was 0.9796 for MDD; *P* < 0.05]. Furthermore, psychiatric disorders also had some causal effects on PDEs [obsessive–compulsive disorder on increased PDE6D and decreased PDE2A and PDE4D; anorexia nervosa on decreased PDE9A]. The results of MR were found to be robust using multiple sensitivity analysis.

**Conclusions:**

In this study, potential causal relationships between plasma PDE proteins and psychiatric disorders were established. Exploring other PDE subtypes not included in this study could provide a more comprehensive understanding of the role of PDEs in psychiatric disorders. The development of specific medications targeting PDE subtypes may be a promising therapeutic approach for treating psychiatric disorders.

**Supplementary Information:**

The online version contains supplementary material available at 10.1186/s12967-023-04368-0.

## Introduction

Phosphodiesterase (PDE) hydrolyzes second messenger molecules [cyclic adenosine monophosphate (cAMP) or cyclic guanosine monophosphate (cGMP)] in cells. The balance between nucleotide cyclase synthesis and hydrolysis inactivation determines the concentration of second messengers [1]. The equilibrium between cAMP and cGMP levels within the nervous system is crucial for learning, memory, and the establishment of neuronal circuits [2, 3]. The levels of both cAMP and cGMP play essential roles in a variety of processes, including axonal development, neurogenesis, axonal neuron polarity, neuronal migration, and synaptic plasticity [2, 4–6]. Also, cAMP and cGMP play significant regulatory roles in cellular activity. Mammalian PDEs are divided into 11 protein subfamilies and are expressed by cells of all tissues [7]. *PDE2A* exhibits significant expression in specific regions of the human brain, namely the frontal, parietal, and temporal cortices. *PDE1A* mRNA is widely expressed throughout the entire human brain [8].

Psychiatric disorder is one of the major public health challenges worldwide, ranking as the second most significant cause of premature death and disability. People with psychiatric disorder have cognitive, emotional or behavioral changes [9, 10]. An increasing number of studies have recently emphasized the significant impact of PDEs on neuropsychiatric disorders [1, 11]. The genetic variations in *PDE* genes may be related to neuropsychiatric disorders, according to genome-wide association studies (GWAS). Rs11976985 in the *PDE1C* gene and rs1513723 in the *PDE7A* gene were found to exhibit associations with autism spectrum disorder (ASD; *P* < 1 × 10^−4^) in a GWAS meta-analysis of more than 16,000 individuals [12]. Informative single-nucleotide polymorphism in *PDE1C* (rs4720058) and *PDE4D* (rs7735958) reached genome-wide significance (*P* < 5 × 10^−8^) with cognitive performance [13]. *PDE2A* mRNA expression dysregulation has been observed in the brain regions associated with the pathophysiology of bipolar disorder (BD), major depressive disorder (MDD), and schizophrenia (SCZ) [14]. Inherited missense variants in the *PDE1B* gene have been identified in probands with SCZ [15]. PDEs have long been recognized as a therapeutic target for a variety of neurological diseases. Recent studies have demonstrated that PDE1 inhibitors can improve both positive and negative symptoms associated with SCZ in animal models [16]. The targeted medications that focus on specific PDE subtypes could serve as a promising therapeutic approach for treating psychiatric disorders. The PDE inhibitors have a positive effect on cognitive function in neurodegenerative conditions such as Alzheimer’s disease (AD) [17, 18]. A double-blind study showed that roflumilast (a PDE4 inhibitor) could enhance verbal memory in patients with SCZ [19]. Clinical studies have shown that PDE4 inhibitors can cross blood–brain barriers and provide benefits for neuroprotection and memory improvement in AD [11, 17]. PDE inhibitors hold great promise as pharmacological agents [7, 18].

Increasing evidence shows that PDEs are associated with psychiatric disorders; however, their cause–effect relationship has not been demonstrated. In these circumstances, the Mendelian randomization (MR) study used genetic variants from GWAS as instrumental variables (IVs) for environmental exposure to draw conclusions about the causality of the result [20]. To this end, a bidirectional two-sample MR study was performed to investigate the causal associations between PDEs and psychiatric disorders.

## Materials and methods

### Study design

**Fig. 1:**
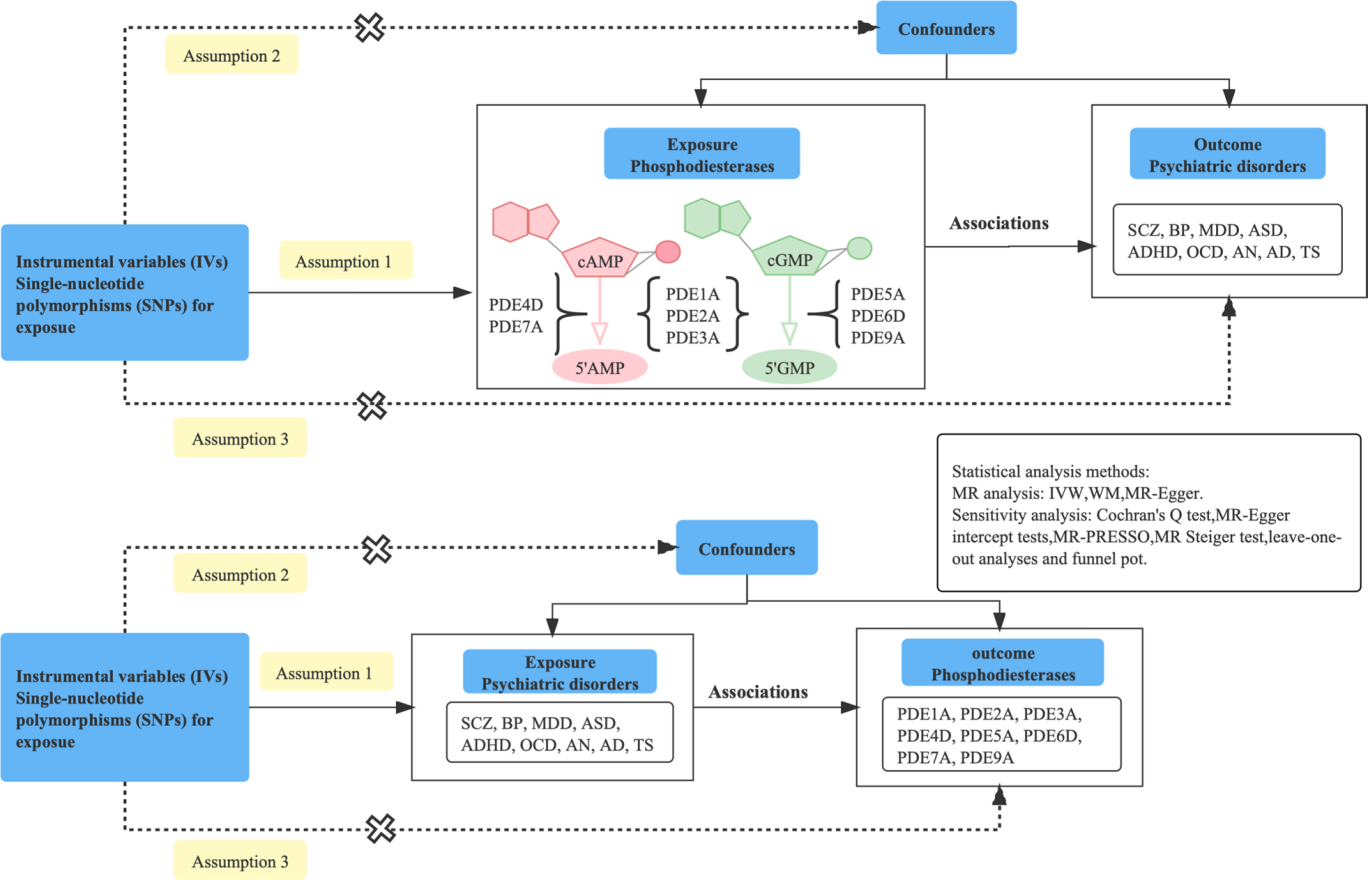
Study flow diagram. The SNPs from GWAS applied as instrumental variables (IVs) were related to exposures (assumption 1). Dash lines with a cross means that IVs must not be associated with confounders (assumption 2) and IVs affect the risk of outcome directly by exposure and not through other alternative pathways (assumption 3). The dash lines indicate irrelevance, and the solid lines indicate relevance. *PDE* phosphodiesterase, *AD* Alzheimer disease, *ADHD* attention-deficit/hyperactivity disorder, *AN* anorexia nervosa, *ASD* autism spectrum disorder, *BD* bipolar disorder, *MDD* major depressive disorder, *OCD* obsessive–compulsive disorder, *SCZ* schizophrenia, *TS* Tourette syndrome, *IVW* inverse-variance weighted, *WM* weighted-median, *MR* Mendelian randomization, *MR-PRESSO* MR pleiotropy residual sum and outlier

A bidirectional MR design was used to detect the causal effects of eight phosphodiesterases (PDEs) on nine psychiatric disorders. This MR analysis was based on three critical assumptions [21] (Fig. 1). A group of PDEs was obtained by hydrolyzing both cAMP and cGMP (PDE1A, PDE2A, and PDE3A), specific to cGMP (PDE5A, PDE6D, and PDE9A) and cAMP (PDE4D and PDE7A). Psychiatric disorders included AD, attention-deficit/hyperactivity disorder (ADHD), anorexia nervosa (AN), ASD, BD, MDD, obsessive–compulsive disorder (OCD), SCZ, and Tourette syndrome (TS).

### Data extraction

Data on the genetic associations were obtained from the Psychiatric Genomics Consortium (PGC: https://www.med.unc.edu/pgc/) and the GWAS summary data (https://gwas.mrcieu.ac.uk/). Only the summarized data of the European population were adopted to reduce the bias of population heterogeneity. Detailed information on the GWAS datasets is provided in Table 1. Informed consent and ethical approval can be found in the original studies.

**Table 1:** Detailed information regarding studies and datasets used in the present study

Exposure or outcome	Preferred names	Source	Ancestry	Participants
Phosphodiesterases				
PDE1A	Dual specificity calcium/calmodulin-dependent 3′,5′-cyclic nucleotide phosphodiesterase 1A	PMID: 29875488	European	3301 individuals
PDE2A	cGMP-dependent 3′,5′-cyclic phosphodiesterase	PMID: 29875488	European	3301 individuals
PDE3A	cGMP-inhibited 3′,5′-cyclic phosphodiesterase 3A	PMID: 29875488	European	3301 individuals
PDE4D	cAMP-specific 3′,5′-cyclic phosphodiesterase 4D	PMID: 29875488	European	3301 individuals
PDE5A	cGMP-specific 3′,5′-cyclic phosphodiesterase	PMID: 28240269	European	997 individuals
PDE6D	Retinal rod rhodopsin-sensitive cGMP 3′,5′-cyclic phosphodiesterase subunit delta	PMID: 29875488	European	3301 individuals
PDE7A	High affinity cAMP-specific 3′,5′-cyclic phosphodiesterase 7A	PMID: 29875488	European	3301 individuals
PDE9A	High affinity cGMP-specific 3′,5′-cyclic phosphodiesterase 9A	PMID: 29875488	European	3301 individuals
Psychiatric disorders				
AD	Alzheimer’s disease	PMID: 30617256	European	71,880 cases and 383,378 controls
ADHD	Attention-deficit/hyperactivity disorder	PMID: 29325848	European	20,183 cases and 35,191 controls
AN	Anorexia nervosa	PMID: 28494655	European	3495 cases and 10,982 controls
ASD	Autism spectrum disorder	PMID: 30804558	European	18,381 cases and 27,969 controls
BD	Bipolar disorder	PMID: 31043756	European	20,352 cases and 31,358 controls
MDD	Major depressive disorder	PMID: 30718901	European	170,756 cases and 329,443 controls
OCD	Obsessive–compulsive disorder	PMID: 28761083	European	2688 cases and 7,037 controls
SCZ	Schizophrenia	PMID: 25056061	European	35,476 cases and 46,839 controls
TS	Tourette syndrome	PMID: 30818990	European	4819 cases and 9488 controls

We extracted the summary statistics of eight PDEs for plasma proteins from two different proteomic GWASs (accessed on 28 December 2022). The genetic predictors for PDE5A were referred from the KORA study with a sample size of 997 individuals [22]. The remaining summary statistics for PDEs (PDE1A, PDE2A, PDE3A, PDE4D, PDE6D, PDE7A, and PDE9A) were obtained from the INTERVAL study with a sample size of 3301 European participants [23]. Summary statistics for nine psychiatric disorders were obtained from the PGC website (accessed on 2 January 2023). The respective sample sizes were as follows: AD [24] (71,880 cases and 383,378 controls), ADHD [25] (20,183 cases and 35,191 controls), AN [26] (3495 cases and 10,982 controls), ASD [27] (18,381 cases and 27,969 controls), BD [28] (20,352 cases and 31,358 controls), MDD [29] (170,756 cases and 329,443 controls), OCD [30] (2688 cases and 7037 controls), SCZ [31] (35,476 cases and 46,839 controls), and TS [32] (4819 cases and 9488 controls) (Table 1).

### Selection of the instrumental variables (IVs)

We selected independent SNPs (*P* value < 1 × 10^−5^) from the GWAS summary data of exposure, which allowed for a sufficient number of SNPs. Also, we collected SNPs at linkage disequilibrium (LD) *r*^2^ threshold < 0.0001 and kb > 10,000 based on the 1000 Genomes European Sample Project [33]. A minor allele frequency threshold of 0.3 was permitted for palindromic SNPs. We used the PhenoScanner V2 database to consider four potential confounders, including drinking, smoking behavior, socioeconomic status and education [34]. In this study, the IVs were not associated with confounders. SNPs with indirect effects were also removed if they were associated (*P* value < 0.001) with the outcome. We calculated the power using the *F* statistics [*F* = *R*^2^ × (N − 2)/(1 − *R*^2^)] for each SNP [35]. Further, we evaluated the *F* statistic values [*F* = ((*R*^2^/(1 − *R*^2^)) × ((N − K − 1)/K)] to assess instrument strength for the MR pairs. Briefly,* N* represents the sample size of the exposure data and the *R*^2^ represents the explained variance of genetic instruments. Based on beta (genetic effect size of the exposure) and SE (standard error of effect size), the F statistic values were also obtained using the formula: *F* = beta^2^/SE^2^ [36]. The general *F* statistic values to measure the power of IV were calculated using 2 methods, with a threshold above 10 implying smaller bias [35].

### Mendelian randomization analyses

Three main MR Methods (Inverse variance weighted (IVW) [37], MR-Egger [38], and weighted median (WM) [37]) were used to investigate the causal relationship between the PDEs and psychiatric disorders. For the main IVW method, the random-effects IVW model was chosen to reduce the influence of heterogeneity on the results [37]. For the reliability of the final analysis results, the following screening criteria were used as filters for robust significant causality: (1) At least one of the three main methods (IVW, MR-Egger, and WM) suggested a significant causal relationship. (2) The direction of MR analysis results (beta value) was consistent among all three methods. (3) We apply the maximum likelihood (ML) method to replicate significant causal relationships, considering our reliance on the IVW method. The ML method is similar to IVW in that it must be presumed beforehand that there is no heterogeneity or horizontal pleiotropy among the IVs. If the IVs meet the assumptions, the results will be unbiased and the standard error will be lesser than with IVW [39]. The Bonferroni correction for multiple testing was conducted to correct *P* values. A *P* value less than 3.47 × 10^−4^ (0.05/72/2; 2 denotes both forward and reverse MR tests) was considered as strong evidence of a causal association. A *P* value less than 0.05 was considered as suggestive evidence for a potential causal association.

### Sensitivity analysis

The sensitivity analysis was conducted to identify any horizontal pleiotropy that would contradict the main MR hypotheses. Thus, we performed MR-Egger intercept tests, Cochran’s Q test, leave-one-out analyses, funnel plot, MR-PRESSO, and MR Steiger test to evaluate the robustness of the results.

The leave-one-out analysis was performed to detect the causal estimates driven by a single SNP. Specifically, the Cochran Q test was used for the IVW model to evaluate the heterogeneity [40]. The MR-Egger regression was used to examine the mean pleiotropic effect of all IVs. The global MR pleiotropy residual sum and outlier (MR-PRESSO) (https://github.com/rondolab/MR-PRESSO/) test was introduced to explore the possible outlier SNPs and detect the presence of horizontal pleiotropy [41]. Additionally, we performed the MR Steiger directionality test to estimate the potential causal association hypothesis between PDEs and psychiatric disorders. MR analyses were conducted using the *TwoSampleMR* package (version 0.5.6) [42].

## Results

### Genetic instruments selected in MR

The study design is shown in Fig. 1. After LD pruning, the outlier IVs based on the funnel plot (Additional file 2: Figs. S1–S18) were also removed for subsequent MR analysis. The details of IVs used in the MR analysis of the association between PDE proteins and psychiatric disorders are provided in Additional file 1: Tables S1 and S2. For the instrument strength for the forward and reverse MR pairs; the *F* statistic values were all ≥ 10 (Additional file 1: Tables S3 and S4).

### Causal effect of genetically predicted PDEs on psychiatric disorders

**Fig. 2:**
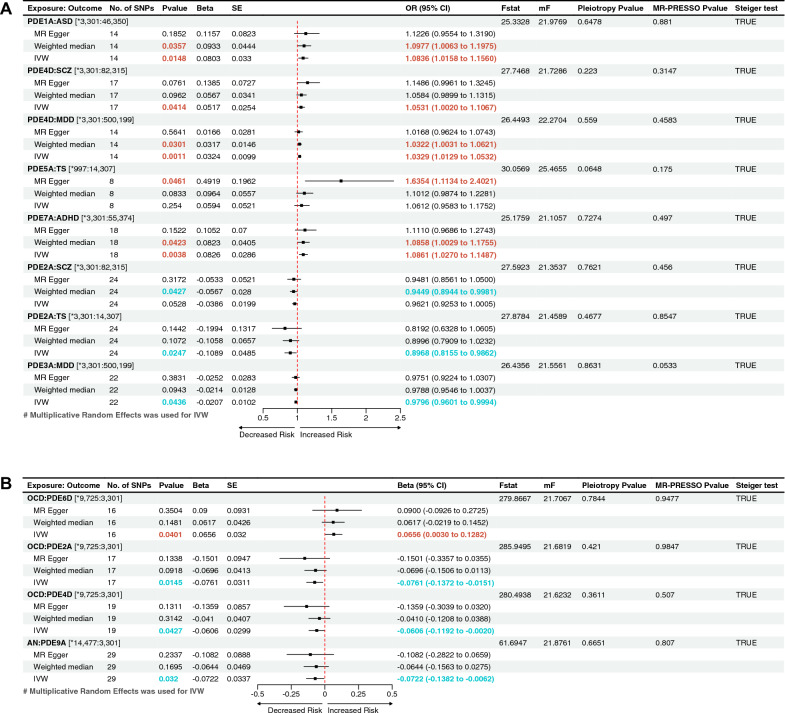
The forest plot shows the significant causalities. Associations between genetically predicted phosphodiesterases (PDEs) and the risk of psychiatric disorders (**A**). Associations between genetically predicted psychiatric disorders and PDEs (**B**). The causal relationships between PDEs and the risk of psychiatric disorders were presented using OR and 95% CIs. Additionally, beta and 95% CIs were used to present the causal relationships between psychiatric disorders and PDEs. Both the pleiotropy p value and the MR-PRESSO p value are greater than 0.05, which means that there is no directional pleiotropy and horizontal pleiotropy. *No* number, *SNP* single nucleotide polymorphism, beta, genetic effect size from the exposure GWAS data, *SE* standard error of effect size, *OR* odds ratio, *CI* confidence interval; F-statistic, [*F* = ((*R*^2^/(1 − *R*^2^)) × ((N − K − 1)/K)]; mF, *F* = beta^2^/SE^2^; Pleiotropy p value, Egger intercept p value; MR, Mendelian randomization; MR-PRESSO p value, MR pleiotropy residual sum and outlier “Global Test” p value. *The sample size of the respective genome-wide association study (GWAS) datasets

**Fig. 3:**
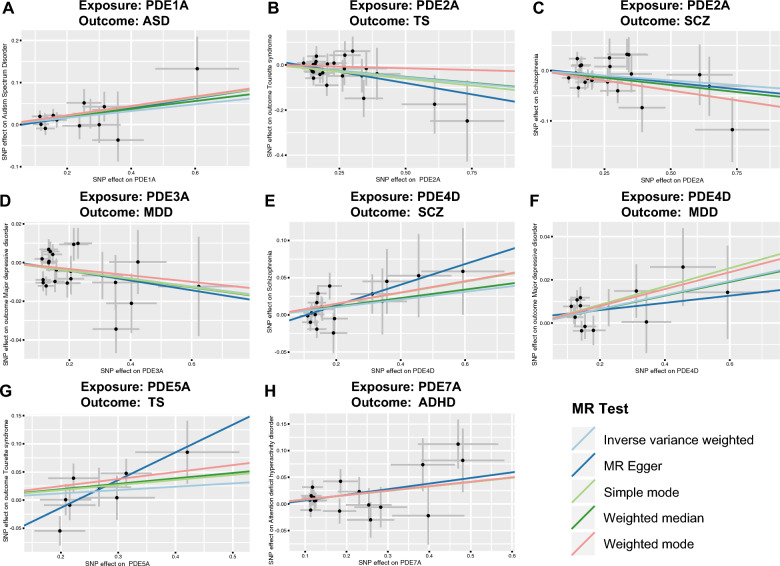
Scatterplot of the effect of the phosphodiesterases (PDEs) on psychiatric disorders. The effect of the PDEs on psychiatric disorders is calculated through instrumental variables (IVs), which provide an association between the PDEs and psychiatric disorders through five Mendelian randomization (MR) methods (**A**–**H**). The slope value equals the b-value calculated using the five methods and represents the causal effect. Positive slope indicates that exposure is a risk factor, whereas a negative slope is the opposite. *PDE* phosphodiesterase, *ASD* autism spectrum disorder, *TS* Tourette syndrome, *SCZ* schizophrenia, *MDD* major depressive disorder, *ADHD* attention-deficit/hyperactivity disorder

The results showed that part of the PDEs was nominally associated with the increased risk of psychiatric disorders (Fig. 2). They were as follows: PDE1A, ASD (odds ratio (OR) = 1.0836, 95% CI 1.0158–1.1560, *P*_IVW_ = 0.0148); PDE4D, SCZ (OR = 1.0531, 95% CI 1.0020–1.1067, *P*_IVW_ = 0.0414); PDE4D, MDD (OR = 1.0329, 95% CI 1.0129–1.0532, *P*_IVW_ = 0.0011); PDE7A, ADHD (OR = 1.0861, 95% CI 1.0270–1.1487, *P*_IVW_ = 0.0038); PDE5A, TS (OR = 1.6354, 95% CI 1.1134–2.4021, *P*_MR-Egger_ = 0.0461). PDE2A was associated with lower-odds SCZ (OR = 0.9449, 95% CI 0.8944–0.9981, *P*_WM_ = 0.0427) and lower-odds TS (OR = 0.8968, 95% CI 0.8155–0.9862; *P*_IVW_ = 0.0247). PDE3A was associated with lower-odds MDD (OR = 0.9796, 95% CI 0.9601–0.9994, *P*_IVW_ = 0.0436). These results of IVW, WM, and MR-Egger tests indicated consistent direction (Fig. 2A). The Bonferroni-corrected threshold (*P* < 3.47 × 10^−4^) served as the statistically significant evidence of a causal association. However, the results provided suggestive evidence of the impact of PDEs on psychiatric disorders. The scatter plots of the effect of PDEs on psychiatric disorders are shown in Fig. 3. The detailed results can be viewed in Additional file 1: Table S5.

### Causal effect of genetically predicted psychiatric disorders on PDEs

**Fig. 4:**
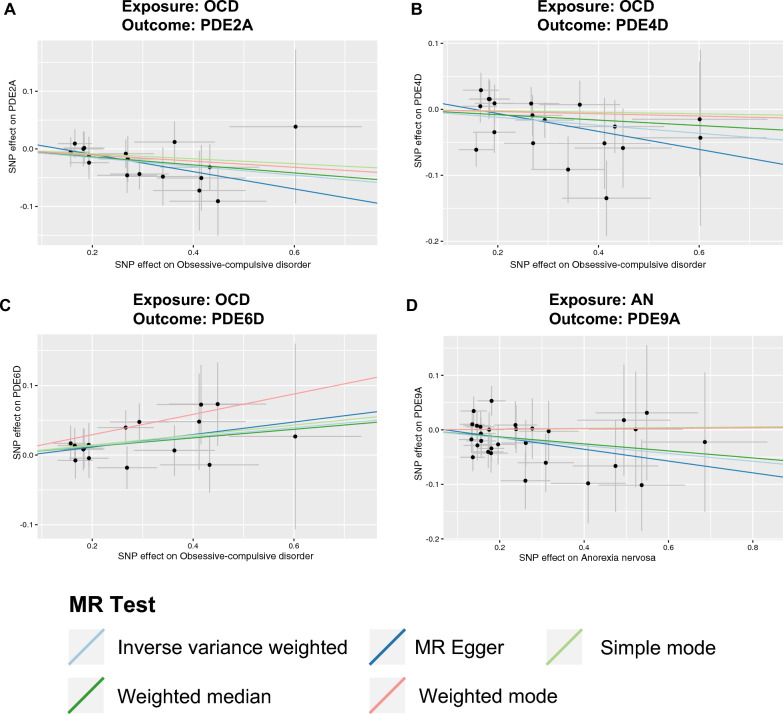
Scatterplot of the effect of the psychiatric disorders on PDEs. An association between the psychiatric disorders on PDEs through five Mendelian randomization (MR) methods (**A**–**D**). The slope value equals the b-value calculated using the five methods and represents the causal effect. Positive slope indicates that exposure is a risk factor, whereas a negative slope is the opposite. *OCD* obsessive–compulsive disorder, *PDE* phosphodiesterase, *AN* anorexia nervosa

The reverse MR analysis was conducted to investigate the putative causal effects of psychiatric disorders on PDEs (Additional file 1: Table S6). A *P* value less than 0.05 indicated suggestive evidence (Fig. 2B). The results showed a nominal causal effect of OCD on increased PDE6D (beta = 0.0656, 95% CI 0.0030–0.1282, *P*_IVW_ = 0.0401), decreased PDE2A (beta = − 0.0761, 95% CI − 0.1372 to − 0.0151, *P*_IVW_ = 0.0145) and decreased PDE4D (beta = − 0.0606, 95% CI − 0.1192 to − 0.0020, *P*_IVW_ = 0.0427). AN was nominally associated with decreased PDE9A (beta = − 0.0722, 95% CI − 0.1382 to − 0.0062, *P*_IVW_ = 0.032). The scatter plots of the effect of PDEs on psychiatric disorders are shown in Fig. 4.

### Sensitivity analyses

We performed sensitivity analyses to verify our putative causalities obtained with bidirectional MR (Table 2). First, as shown by the funnel plot, the effect size variation around the point estimate was symmetrical after excluding outliner SNPs (Additional file 2: Figs. S1–S18). The MR-PRESSO test provided no evidence of possible outliers. Second, all P values were > 0.05 in the MR-PRESSO global tests, Cochran’s Q tests, and the MR-Egger intercept tests, manifesting no evidence of heterogeneity and horizontal pleiotropy. Third, the “leave-one-out” method confirmed that single SNPs did not affect the causal association (Additional file 2: Figs. S19–S30). Fourth, the directions of the estimates from the WM and MR-Egger tests were the same as those from the IVW method.

**Table 2:** Sensitivity analysis of the causal association between PDEs proteins and psychiatric disorders

Exposure: outcome	MR-Eegger_Intercept	Egger_intercept_pval^a^	IVW_Cochrane_Q	IVW_Cochrane_Q_pval^b^	MR-PRESSO Pvalue^c^	Steiger_test^d^
PDE1A: ASD	− 0.0069	0.6478	7.6507	0.8656	0.8810	TRUE
PDE2A: SCZ	0.0031	0.7621	23.5793	0.4274	0.4560	TRUE
PDE2A: TS	0.0190	0.4677	15.6579	0.8695	0.8547	TRUE
PDE3A: MDD	0.0008	0.8631	33.4772	0.0412	0.0533	TRUE
PDE4D: MDD	0.0027	0.5590	13.0823	0.4415	0.4583	TRUE
PDE4D: SCZ	− 0.0148	0.2230	18.0200	0.3227	0.3147	TRUE
PDE5A: TS	− 0.1120	0.0648	10.8504	0.1453	0.1750	TRUE
PDE7A: ADHD	− 0.0039	0.7274	16.6441	0.4787	0.4970	TRUE
OCD: PDE2A	0.0205	0.4210	6.1782	0.9861	0.9847	TRUE
OCD: PDE4D	0.0208	0.3611	18.0066	0.4552	0.5070	TRUE
OCD: PDE6D	− 0.0065	0.7844	6.8627	0.9613	0.9477	TRUE
AN: PDE9A	0.0077	0.6651	21.5561	0.8014	0.8070	TRUE
AD: PDE7A	− 0.0022	0.6965	67.5274	0.4247	0.4727	FALSE

*Q* Cochran’s Q statistics, *IVW* inverse-variance weighted, *MR* Mendelian randomization, *MR-PRESSO* MR pleiotropy residual sum and outlier, *PDE* phosphodiesterase, *ASD* autism spectrum disorder, *SCZ* schizophrenia, *TS* Tourette syndrome, *MDD* major depressive disorder, *ADHD* attention-deficit/hyperactivity disorder, *OCD* obsessive–compulsive disorder, *AN* anorexia nervosa, *AD* Alzheimer’s disease

^a^The MR–Egger intercept quantifies the effect of directional pleiotropy (*P* < 0.05, which means possible directional pleiotropy)

^b^The Cochrane—Q test quantifies the effect of heterogeneity (P < 0.05, which means possible heterogeneity, thus prioritizing “random—IVW” methods)

^c^MR-PRESSO test quantifies the effect of horizontal pleiotropy (*P* < 0.05, which means possible horizontal pleiotropy)

^d^MR-Steiger directionality test to assess the potential causal relationship (FALSE, which means an inverse causal link)

Additionally, the MR Steiger test was used to detect the reliability of the causal direction. The Steiger test result between AD and PDE7A was FALSE, suggesting an inverse causal link (Table 2). However, the results of the MR Steiger test supported our conclusions regarding 12 potential causal relationships between PDEs and psychiatric disorders (Fig. 2).

Using the ML method, we replicated the vast majority of significant causal relationships (*P*_ML_ < 0.05), except for the relationship from PDE5A to TS (*P*_ML_ = 0.241). This result supports the robustness of our analysis of the causal relationship between PDEs and psychiatric disorders, avoiding the occurrence of accidental errors. The Additional file 2: Fig. S31 shows the results of the re-MR analysis using the ML method.

## Discussion

In the present study, we performed bidirectional MR analyses to systematically evaluate the causal associations between eight PDEs and nine psychiatric disorders. The forward MR analysis showed that genetically predicted PDEs specific to cAMP were associated with higher-odds psychiatric disorders. For example, PDE4D was associated with higher odds of SCZ and MDD, while PDE7A was associated with higher odds of ADHD. Additionally, suggestive evidence for PDE1A (which hydrolyzes both cAMP and cGMP) on ASD was obtained. We observed a negative association of PDE2A with SCZ, PDE2A with TS, and PDE3A with MDD. The reverse MR analysis showed that OCD was associated with increased PDE6D, and decreased PDE2A and PDE4D. AN was associated with decreased PDE9A.

The observational studies reported that PDEs were associated with psychiatric disorders. This was the first MR study to estimate the causal association between PDEs and psychiatric disorders. MR examined causality with the large-scale GWAS data using genetic polymorphisms as a proxy for exposure [43]. The suggestive causal associations were found between PDEs and psychiatric disorders. Furthermore, the consistency across sensitivity analyses further reinforces the credibility of the effect estimates. The intracellular concentrations of cyclic nucleotides are regulated by PDE hydrolases that change cAMP and cGMP into 5′AMP and 5′GMP [1]. These associations suggest that the dysregulation between PDEs and cAMP/cGMP signaling as a potential cause of psychiatric disorders. The improved effects of PDE activity regulation on cognitive symptoms and depressive behavior have also gained attention, supporting the notion that PDEs play a role in the pathophysiology and pharmacotherapy of psychosis [18, 44]. The pharmacological effects of PDE inhibitors have also been investigated [44], and the clinical trials for PDE target-specific drugs are still ongoing [18].

In this study, PDE4D protein was positively correlated with both the risk of SCZ and the risk of MDD. Our findings provided evidence that PDE4 was a potential therapeutic drug target. PDE4 is an isoenzyme with multiple isoforms, which is widely expressed in a variety of tissues and primarily hydrolyzes cAMP. Previously studied antipsychotics can increase intracellular cAMP levels by antagonizing neurotransmitter receptors [45]. Inhibition of PDE4 can increase intracellular cAMP levels while functionally salvaging synaptic defects [46]. The activity of PDE regulates cAMP response element–binding protein (CREB) and cAMP-activating protein kinase A (PKA) [47]. Numerous studies on animals have demonstrated the potent antidepressant effects of PDE4 inhibitors [44, 48]. Rolipram, a PDE4 inhibitor, increases brain-derived neurotrophic factor (BDNF) expression through cAMP/CREB and exerts antidepressant effects [49, 50]. The genetic factors disrupted in schizophrenia 1 (DISC1) and PDE4 collaborate to regulate cAMP signaling in schizophrenia [46, 51]. Roflumilast (a PDE4 inhibitor) can enhance verbal and working memory in patients with SCZ [19]. The second messengers, cAMP and cGMP, influence a wide range of physiological processes, including neurotransmitter signaling, inflammation, molecular signal transduction, and the transcription of many genes [2, 52, 53]. Significantly, a majority of PDEs, which belong to an enzyme family that controls cAMP and cGMP levels, are found in the nervous system and all neurons [54].

PDE7 inhibitors promote neural differentiation and neuroprotection by activating the cAMP/PKA signaling [55]. In this study, PDE7A was associated with an increased risk of ADHD. Inhibiting PDE7A improves cAMP/CREB signaling, encourages the differentiation of neural stem cells, and improves memory and learning, according to rodent model studies [56, 57]. PDE7A and PDE7B play a role in regulating dopaminergic signaling and are primarily expressed in the striatum. Studies have also revealed the therapeutic potential of S14, a small-molecule inhibitor of PDE7, in treating neurodegenerative diseases, particularly Parkinson’s disease [57]. PDE7 is a new potential immunopharmacological anti-inflammatory target for treating chronic inflammation and neurodegenerative diseases [58]. Exploring specific PDE isoforms and increase intracellular cAMP levels can deepen our understanding and inform the development of targeted interventions.

Psychiatric medications have shown inconsistent responses to the cAMP cascade. Previous study demonstrated that haloperidol increased cAMP levels, chronic usage of clozapine has been found to decrease cAMP levels [59]. The forward MR analysis identified PDE1A as a risk factors for ASD, while suggesting potential protective effects of PDE2A in SCZ and TS. The genetic variations in *PDE1C* and *PDE7A* were found to be associated with ASD in the GWAS study, which examined 7387 cases of ASD and 8567 controls [12]. Decreased *PDE2A* mRNA levels were found in BD and SCZ, with changes most pronounced in the frontal cortical regions of SCZ patients and the hippocampus and striatum of BD patients [14]. A link between SCZ and MDD and the levels of the PDE2A protein was not established in this study using reverse MR analysis. Homozygous mutations in *PDE2A* have been shown to be associated with neurodevelopmental and intellectual disability [60, 61]. This study found that alterations in PDE protein levels were associated with OCD and AN, but they were not identified as risk or protective factors. In association studies of Chinese populations, rs1838733 in the *PDE4D* gene was found to be associated with OCD [62]. However, more research is needed to fully understand the role of PDEs in psychiatric disorders and to determine whether targeting this enzyme could be a viable treatment option for individuals with the disorder. PDE tracers can allow specific changes in different brain regions to be observed by PET imaging in vivo, while nanomedicines can target and release drugs [63–65]. There is potential to evaluate potential PDE drugs for different psychiatric disorders in the future and combine them with new technologies. Much directed laboratory and clinical studies in humans are needed to fully understand the impact of PDE subtypes in psychiatric disorders.

The present study had several advantages. First, nine psychiatric disorders and eight PDEs were included, making it the first comprehensive MR study on the association between the PDE system and psychiatric disorders. Second, multiple sensitivity analyses provided evidence that the assumed causal effect in our MR results was reliable. However, this study also had some limitations. First, the *P*-value threshold was set at 1 × 10^−5^ to ensure that sufficient SNPs were included to maintain the study power. The study had no weak IVs according to the *F* statistics. The GWAS studies included in this research are based on European populations. Second, other PDEs that were not included in the analysis might also have importance in psychiatric disorders. Further, the potential causal associations reported in this study should be interpreted with caution, given that the *P* values were almost at nominal levels. Third, MR analysis uses exposure risk SNPs to examine the impact of lifetime exposure on outcomes, and the effect sizes of MR analyses may be different from those of randomized controlled trials of short-term interventions. To address the limitations mentioned, larger and more diverse datasets, conduct replication studies are required to fully comprehend the genetic implications on exposures. And future research could include populations with different characteristics (such as race and age) in MR studies may increase the representation of different populations. Exploring other PDE subtypes not included in this study could provide a more comprehensive understanding of the role of PDEs in psychiatric disorders. Furthermore, a multidisciplinary approach involving functional studies, genetic investigations and clinical trials can investigate the mechanisms through which PDEs influence psychiatric disorders could offer insights into potential therapeutic targets and pathways.

## Conclusions

In conclusion, this bidirectional MR study provided additional insights into the relationships between PDEs and psychiatric disorders. Our findings implied that PDEs and cAMP/cGMP defense might be useful in etiological research and personalized medicine in psychiatric disorders. The PDEs are potential candidates as novel drug targets for psychiatric disorders.

### Supplementary Information

**Additional file 1: Table S1.** Instrumental variables used in MR analysis of the association between PDEs protein and psychosis. **Table S2.** Instrumental variables used in MR analysis of the association between psychosis and PDEs protein. **Table S3.** Sensitivity analysis of the causal association between PDEs protein on psychosis. **Table S4.** Sensitivity analysis of the causal association between psychosis on PDEs protein. **Table S5.** Mendelian Randomization estimation for PDEs on the risk of psychosis. **Table S6.** Mendelian Randomization estimation for psychosis on the risk of PDEs.

**Additional file 2: Figures S1–S30.** The Funnel plot and Leave-one-out analysis between PDEs protein and psychosis. **Figure S31.** The forest plot shows the significant causalities by maximum likelihood (ML) methods.

## Data Availability

The original contributions presented in the study are included in the article/Additional files. The code for this article can be found online at: https://github.com/YWH199310/MR_-process_code, further inquiries can be directed to the corresponding authors.

## Abbreviations

AD: Alzheimer’s disease
ADHD: Attention-deficit/hyperactivity disorder
AN: Anorexia nervosa
ASD: Autism spectrum disorder
BD: Bipolar disorder
cAMP: Cyclic adenosine monophosphate
cGMP: Cyclic guanosine monophosphate
IVW: Inverse variance weighted method
MDD: Major depressive disorder
MR: Mendelian randomization
OCD: Obsessive–compulsive disorder
OR: Odds ratios
PDE: Phosphodiesterase
SCZ: Schizophrenia
SNP: Single nucleotide polymorphism
TS: Tourette syndrome
