# Supplementary material for: Phosphodiesterase and psychiatric disorders: a two-sample Mendelian randomization study
Source: J Transl Med. 2023 Aug 21;21:560. doi: 10.1186/s12967-023-04368-0 (PMC10441701; doi:10.1186/s12967-023-04368-0)
Supplement: Supplementary file 2 — Additional file 2: Figures S1–S30. The Funnel plot and Leave-one-out analysis between PDEs protein and psychosis. Figure S31. The forest plot shows the significant causalities by maximum likelihood (ML) methods. [file 12967_2023_4368_MOESM2_ESM.docx]

Additional Figures

Figure S1. Funnel plot for PDE1A on Autism spectrum disorder.

Figure S2. Funnel plot for PDE2A on Tourette syndrome.

Figure S3. Funnel plot for PDE2A on Schizophrenia.

Figure S4. Funnel plot for PDE3A on Major depressive disorder.

Figure S5. Funnel plot for PDE4D on Schizophrenia.

Figure S6. Funnel plot for PDE4D on Major depressive disorder.

Figure S7. Funnel plot for PDE5A on Tourette syndrome.

Figure S8. Funnel plot for PDE7A on Attention deficit hyperactivity disorder.

Figure S9. Funnel plot for Obsessive-compulsive disorder on PDE2A.

Figure S10. Funnel plot for Obsessive-compulsive disorder on PDE4D.

Figure S11. Funnel plot for Obsessive-compulsive disorder on PDE6D.

Figure S12. Funnel plot for Anorexia nervosa on PDE9A.

Figure S13. Funnel plot from genetically predicted PDE2A on Major depressive disorder.

Figure S14. Funnel plot from genetically predicted PDE3A on Schizophrenia.

Figure S15. Funnel plot from genetically predicted PDE4D on Tourette syndrome.

Figure S16. Funnel plot from genetically predicted PDE9A on Attention deficit hyperactivity disorder.

Figure S17. Funnel plot from genetically predicted PDE9A on Alzheimer's disease.

Figure S18. Funnel plot from genetically predicted Alzheimer's disease on PDE9A.

Figure S19. Leave-one-out analysis for PDE1A on Autism spectrum disorder.

Figure S20. Leave-one-out analysis for PDE2A on Tourette syndrome.

Figure S21. Leave-one-out analysis for PDE2A on Schizophrenia.

Figure S22. Leave-one-out analysis for PDE3A on Major depressive disorder.

Figure S23. Leave-one-out analysis for PDE4D on Schizophrenia.

Figure S24. Leave-one-out analysis for PDE4D on Major depressive disorder.

Figure S25. Leave-one-out analysis for PDE5A on Tourette syndrome.

Figure S26. Leave-one-out analysis for PDE7A on Attention deficit hyperactivity disorder.

Figure S27. Leave-one-out analysis for Obsessive-compulsive disorder on PDE2A.

Figure S28. Leave-one-out analysis for Obsessive-compulsive disorder on PDE4D.

Figure S29. Leave-one-out analysis for Obsessive-compulsive disorder on PDE6D.

Figure S30. Leave-one-out analysis for Anorexia nervosa on PDE9A.

**Figure S31.** **The forest plot shows the significant causalities by maximum likelihood (ML) methods.**

Additional Figures


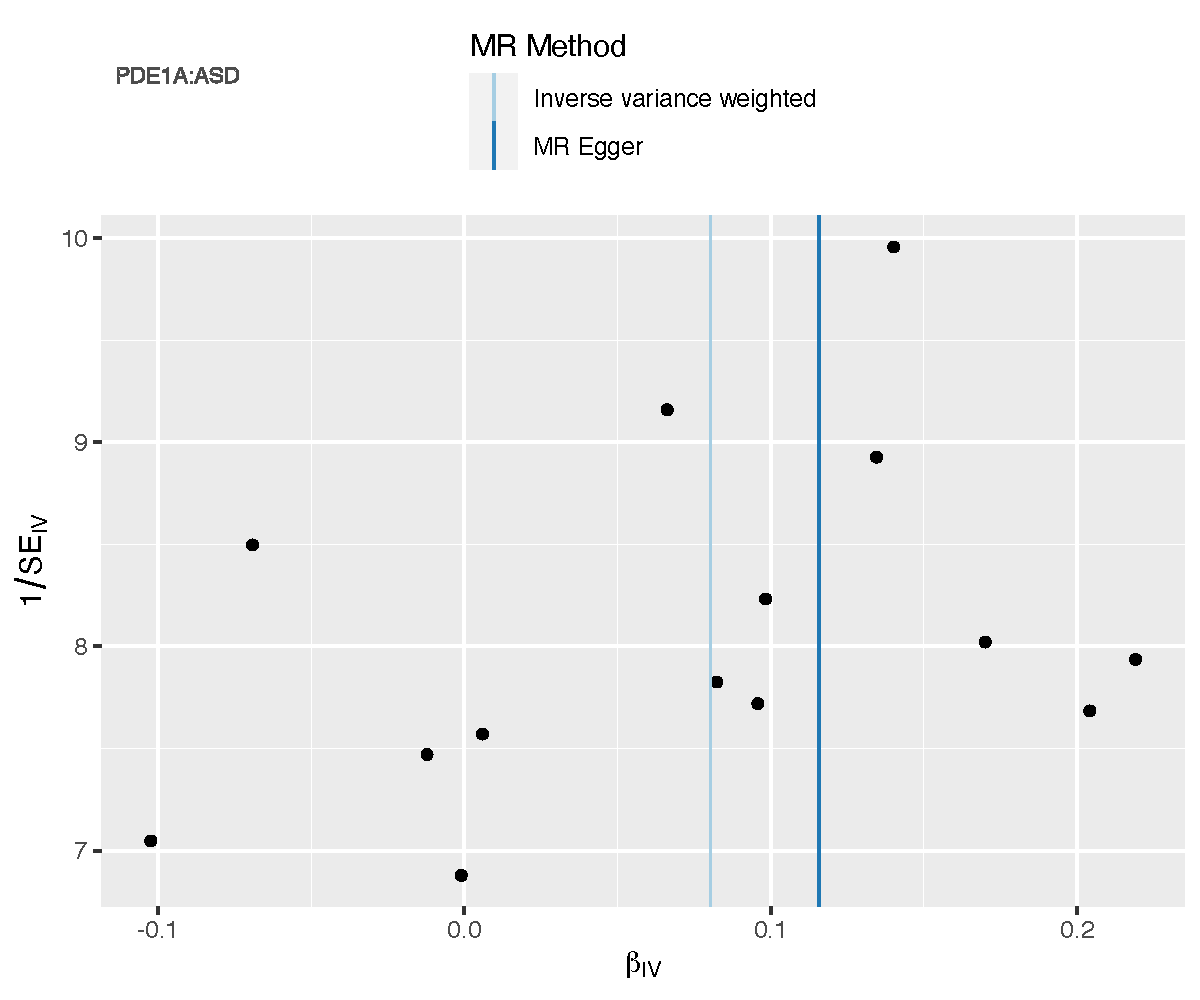


**Figure S1.** Funnel plot for PDE1A on Autism spectrum disorder.


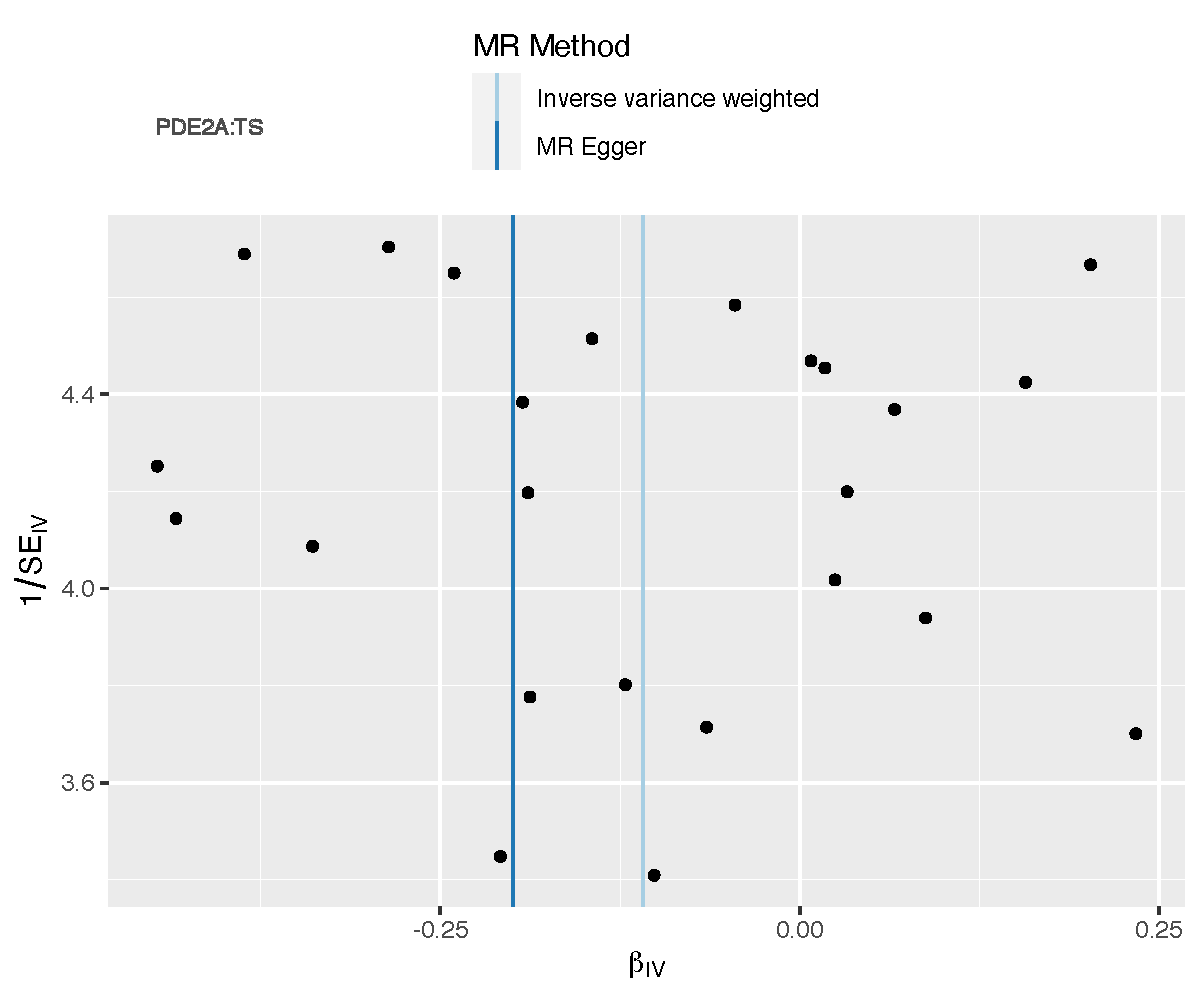


**Figure S2.** Funnel plot for PDE2A on Tourette syndrome.


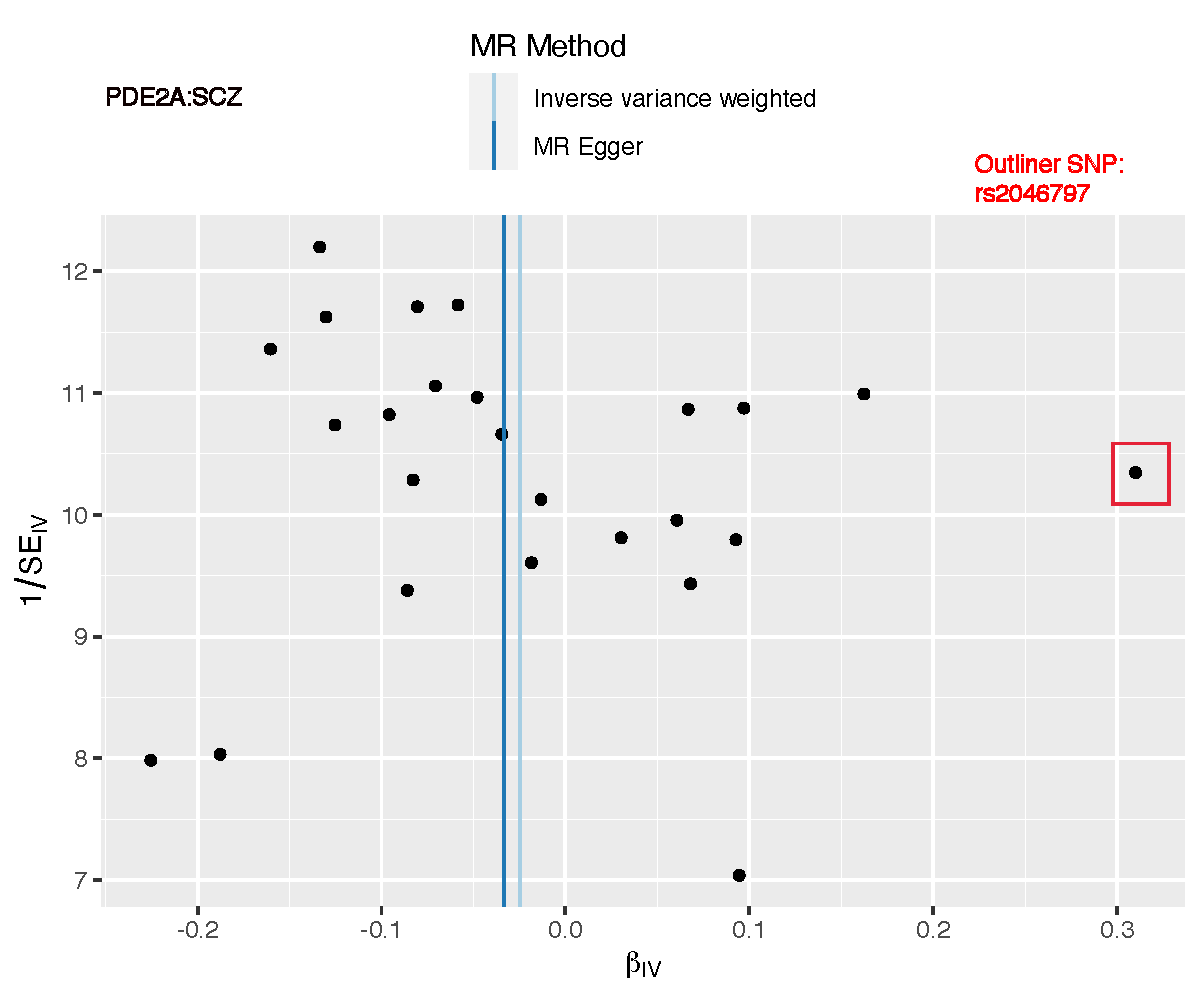


**Figure S3.** Funnel plot for PDE2A on Schizophrenia.


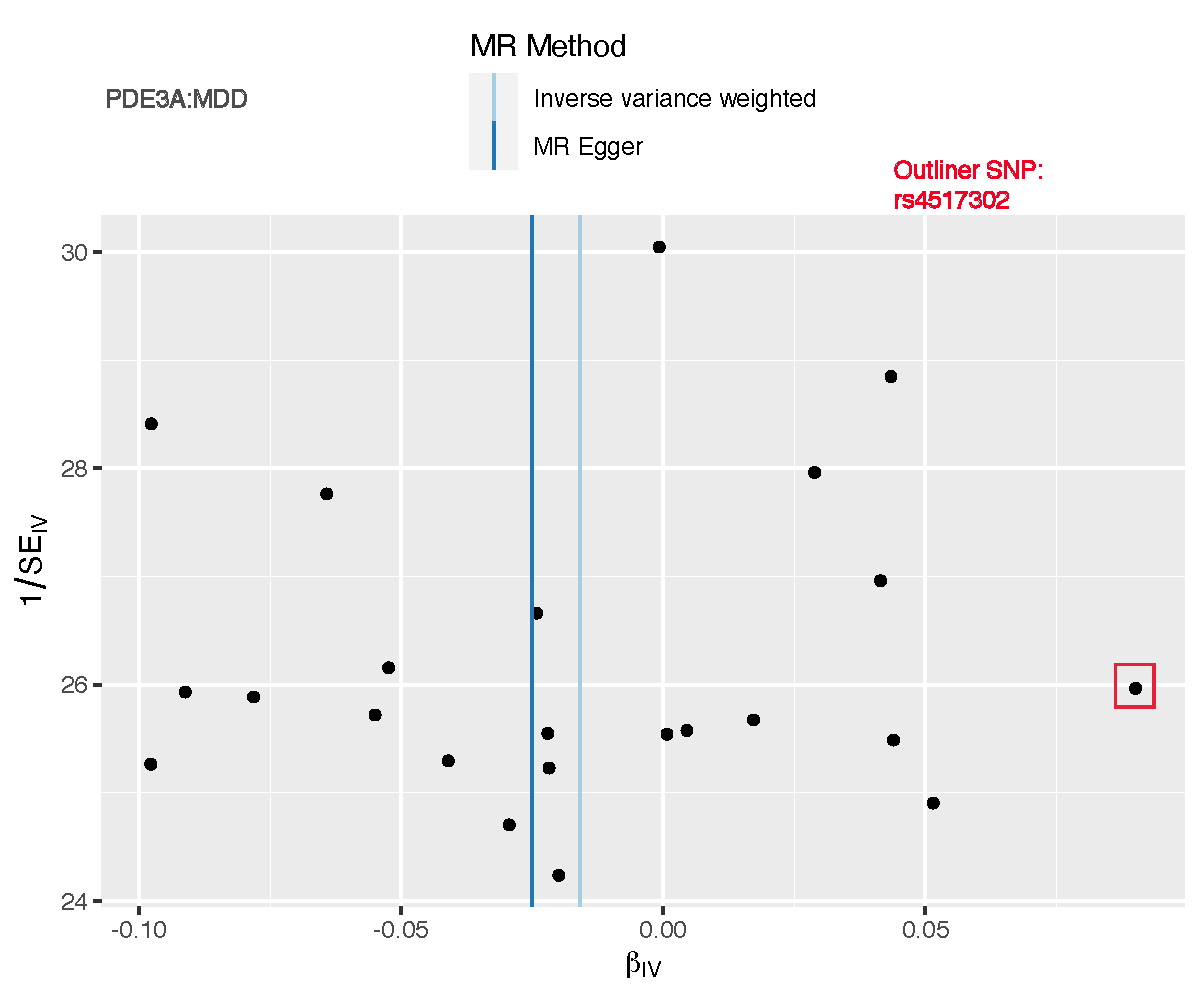


**Figure S4.** Funnel plot for PDE3A on Major depressive disorder.


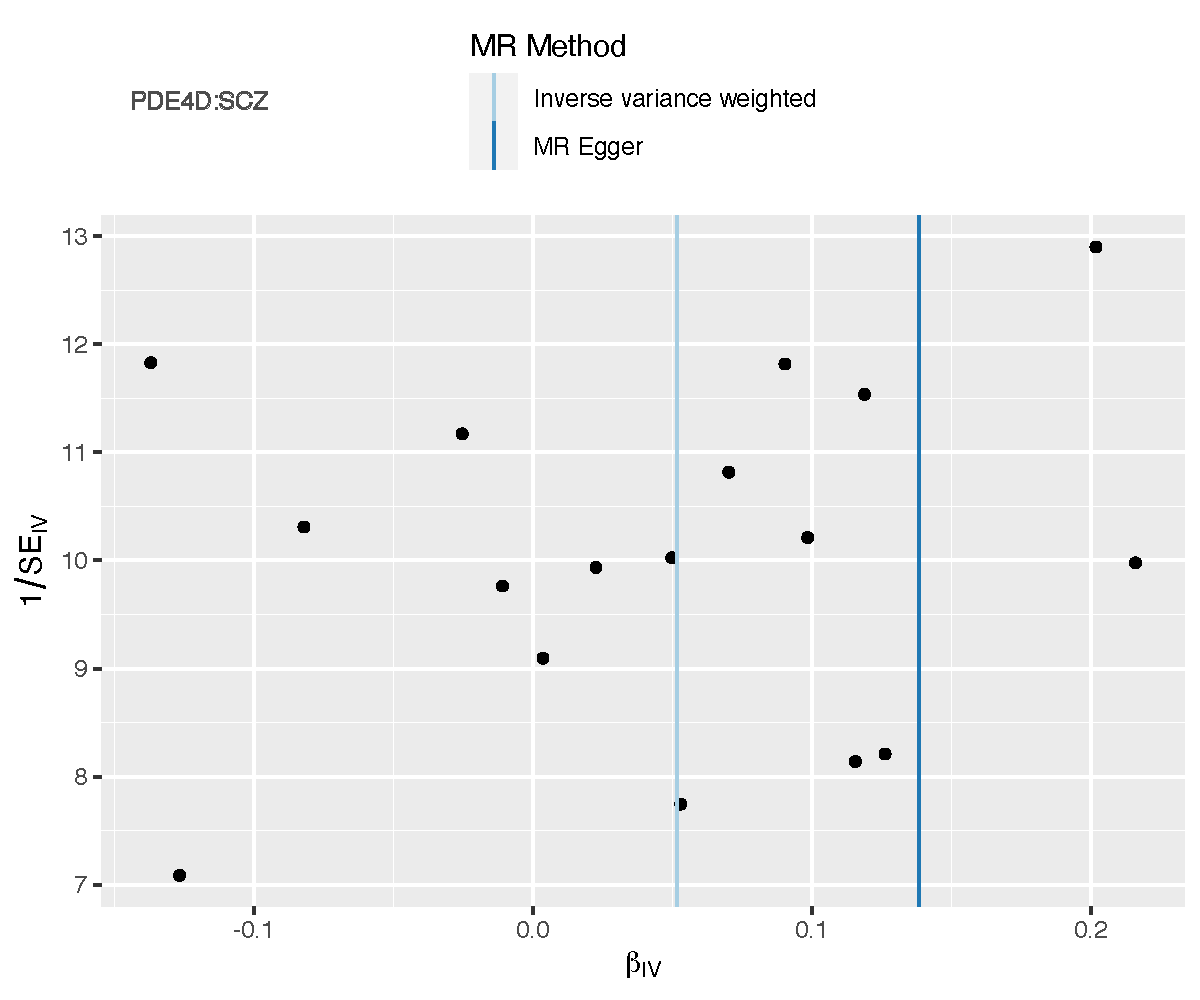


**Figure S5.** Funnel plot for PDE4D on Schizophrenia.


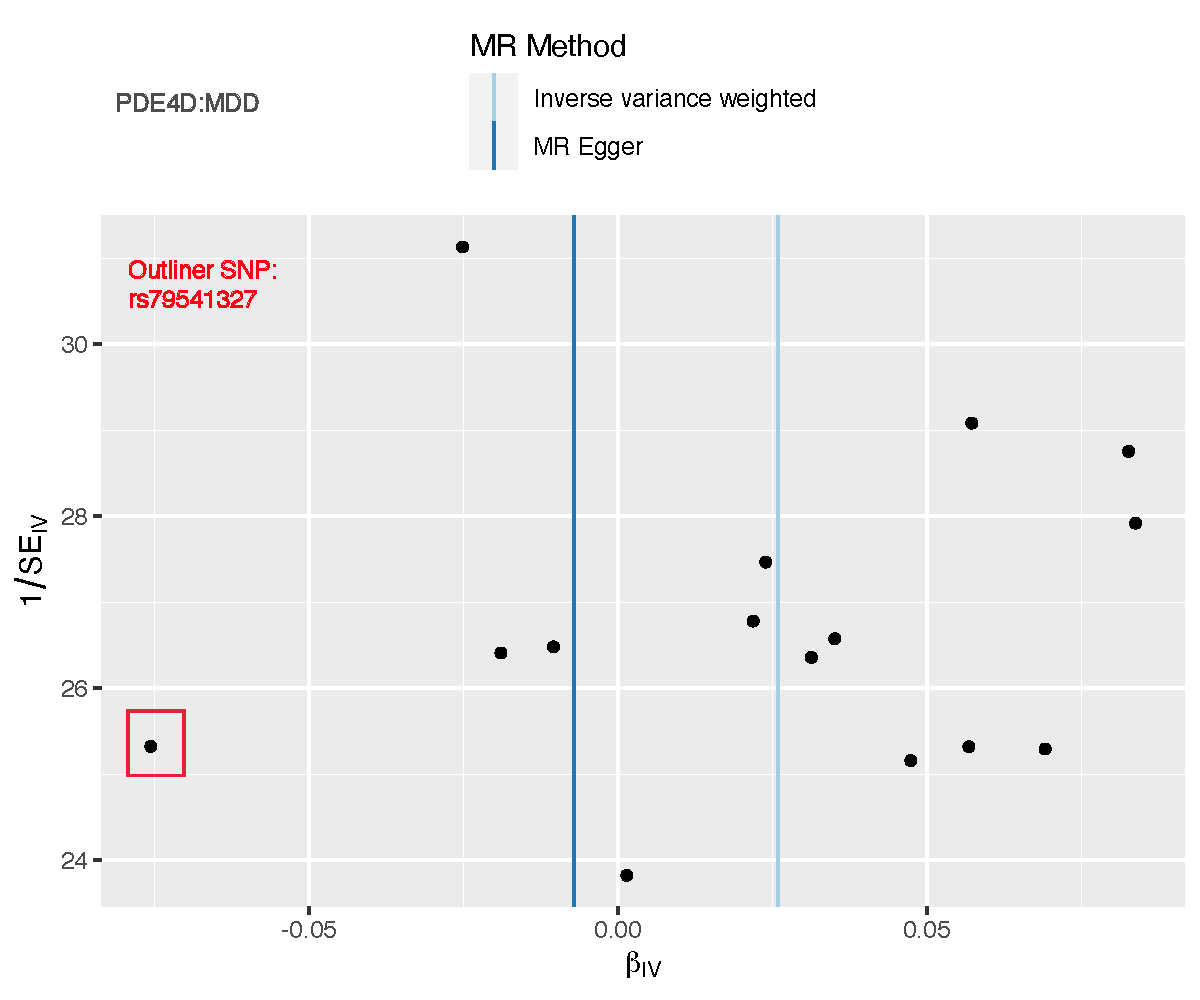


**Figure S6.** Funnel plot for PDE4D on Major depressive disorder.


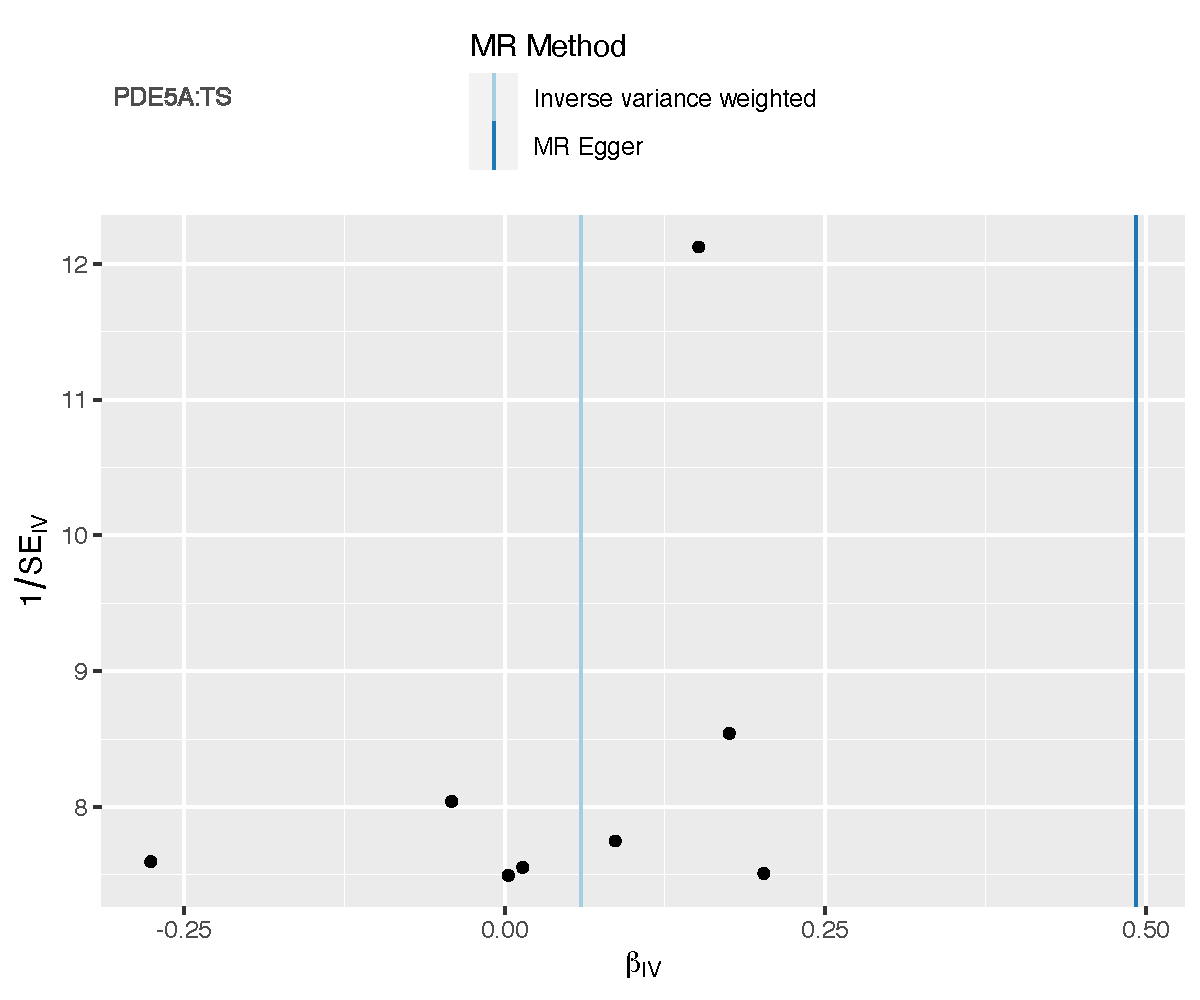


**Figure S7.** Funnel plot for PDE5A on Tourette syndrome.


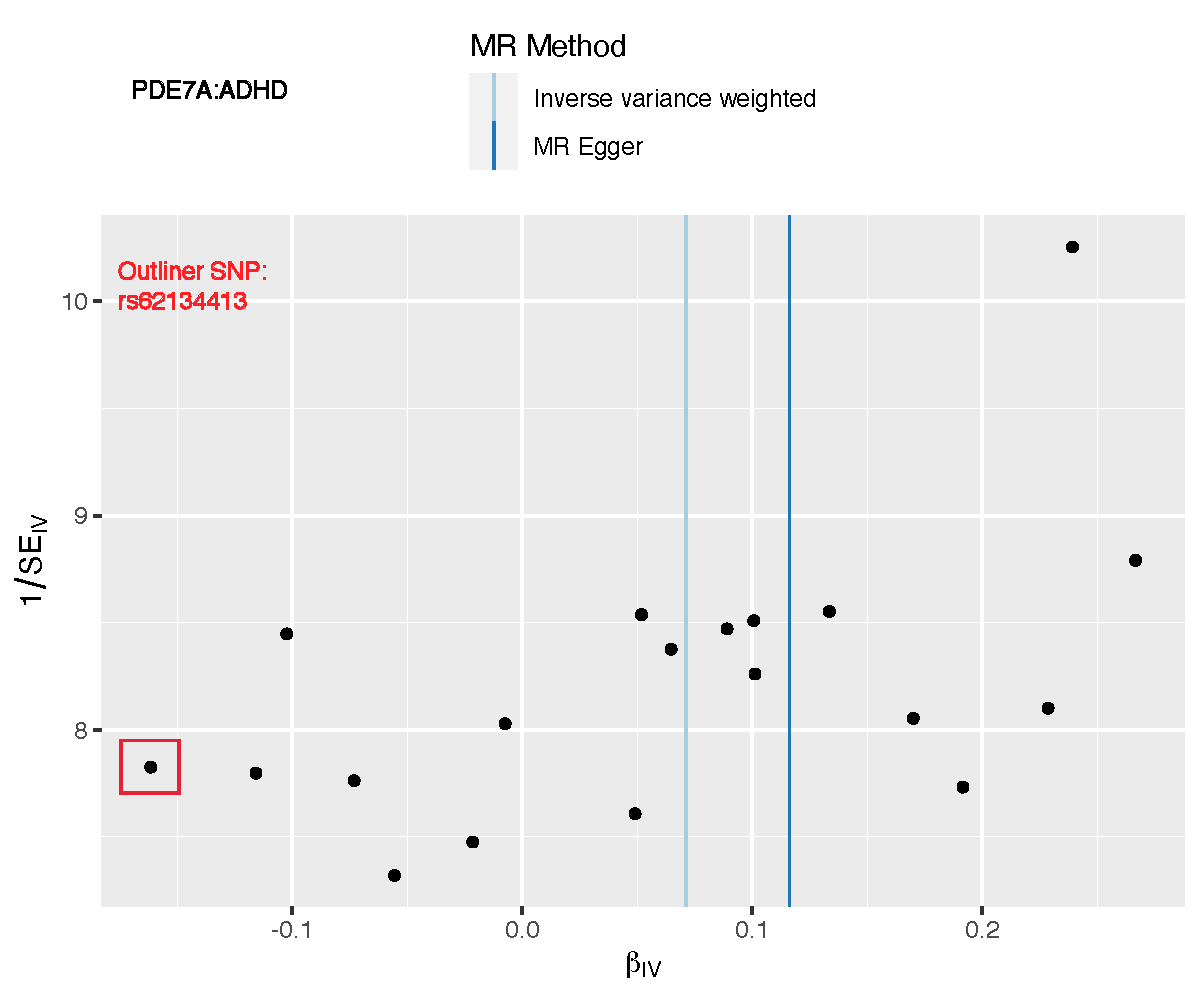


**Figure S8.** Funnel plot for PDE7A on Attention deficit hyperactivity disorder.


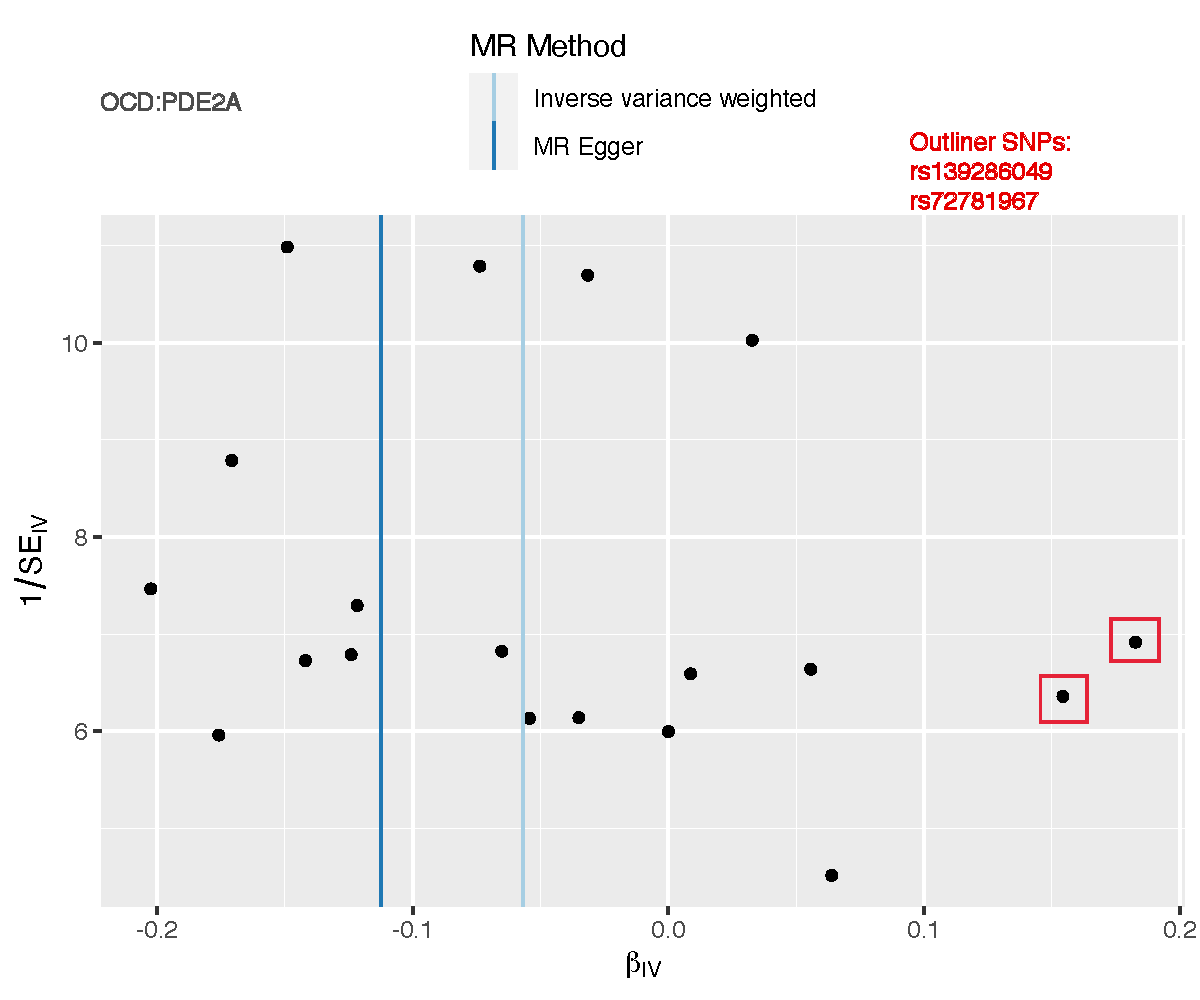


**Figure S9.** Funnel plot for Obsessive-compulsive disorder on PDE2A.


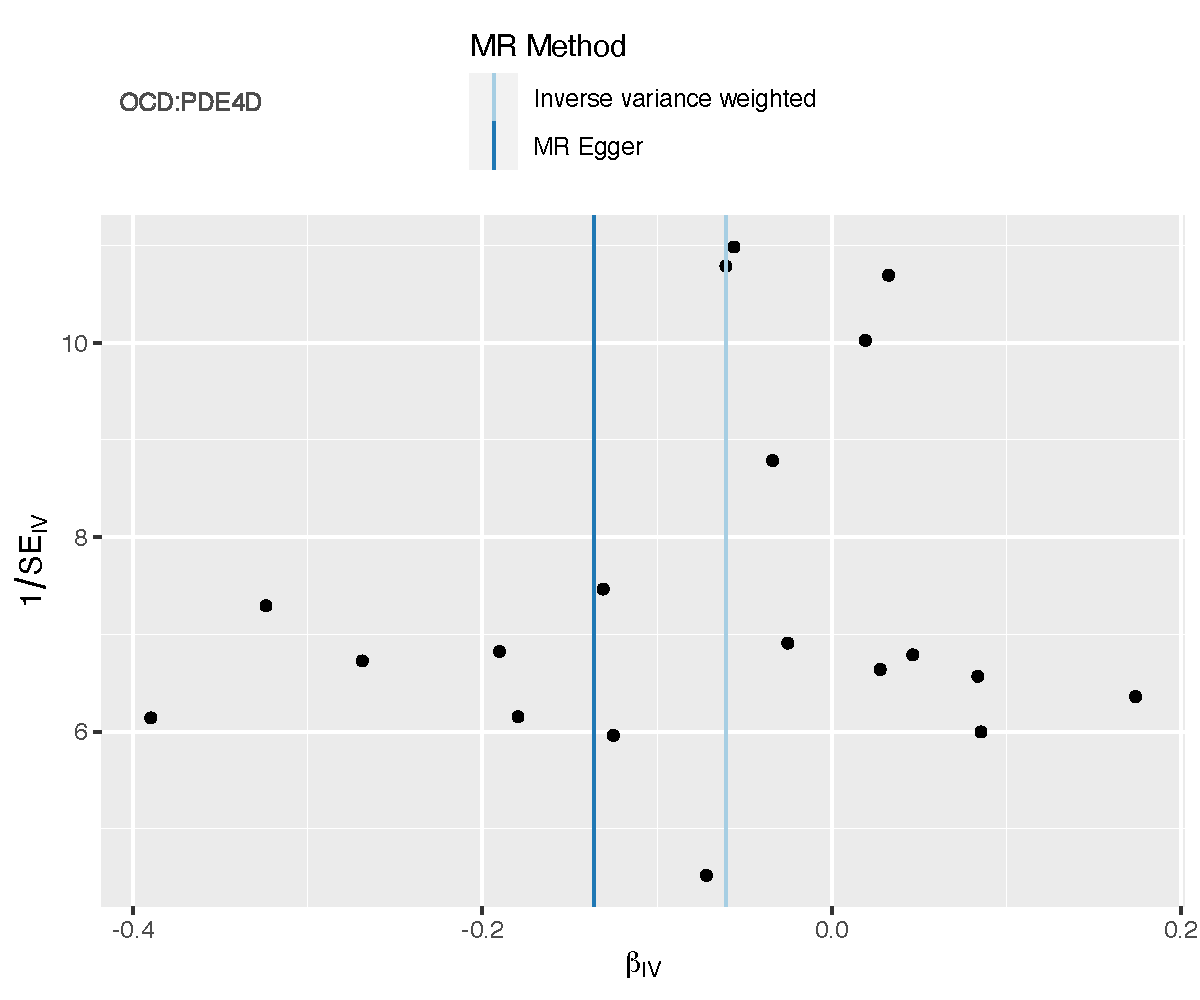


**Figure S10.** Funnel plot for Obsessive-compulsive disorder on PDE4D.


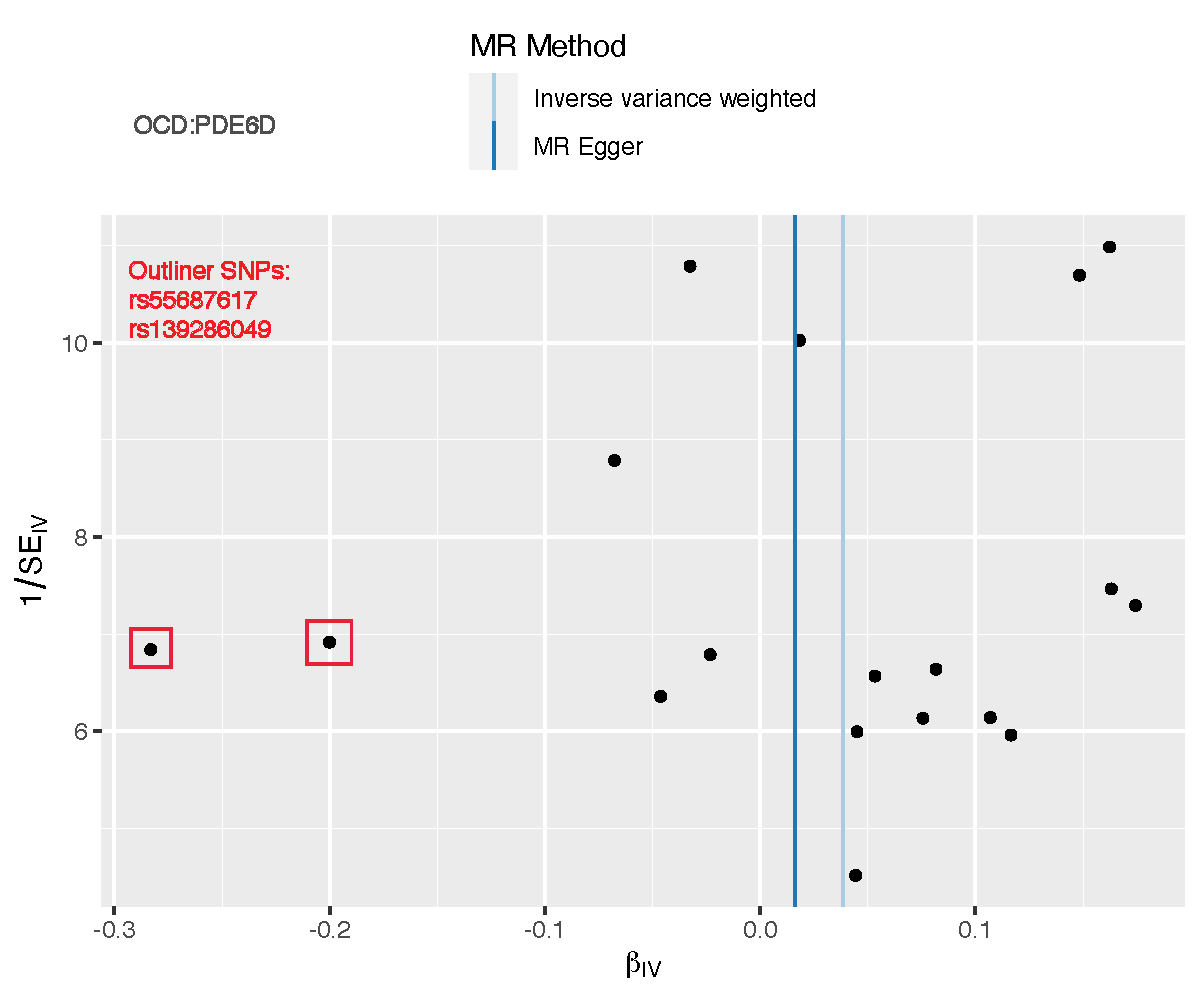


**Figure S11.** Funnel plot for Obsessive-compulsive disorder on PDE6D.


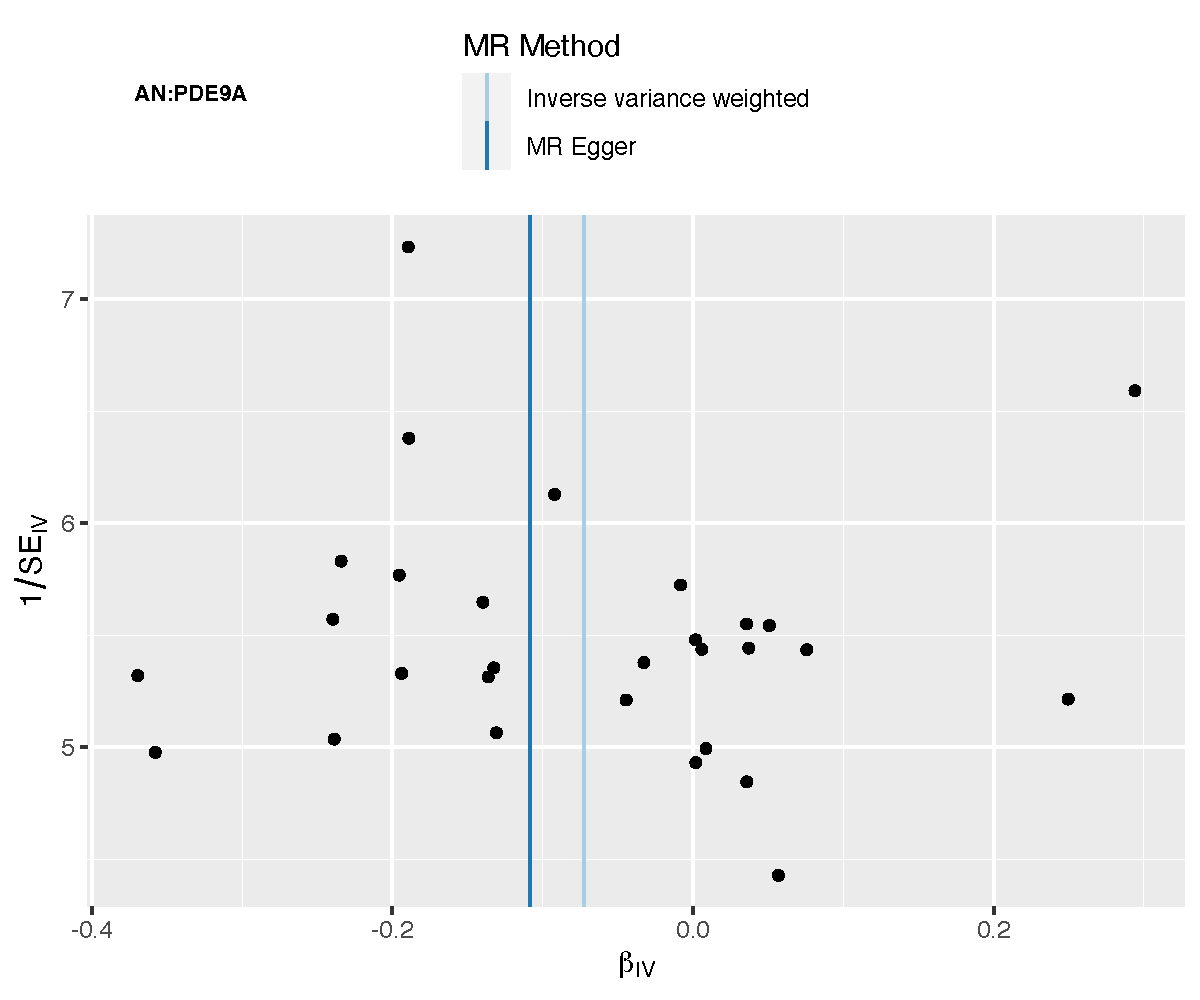


**Figure S12.** Funnel plot for Anorexia nervosa on PDE9A.


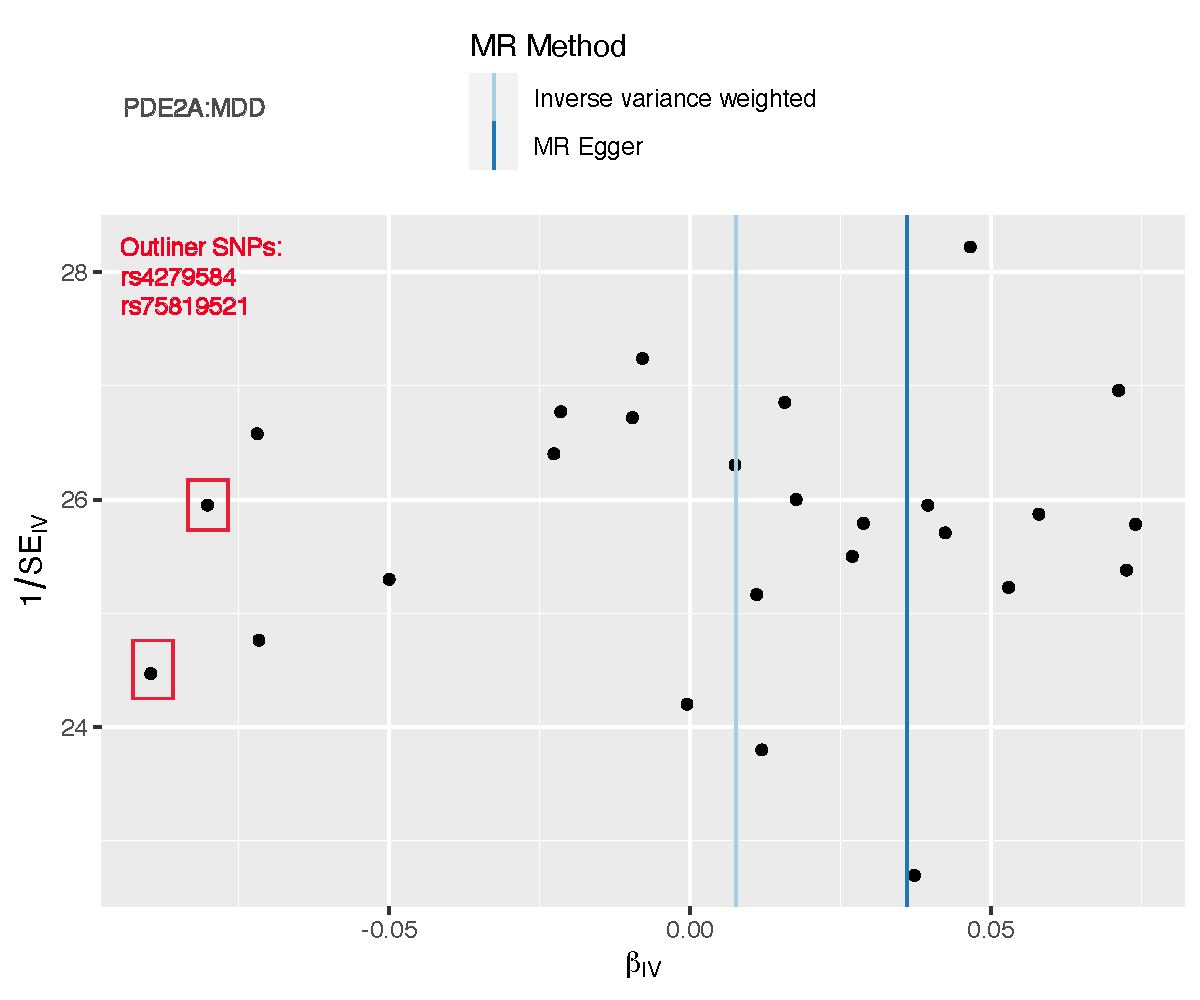


**Figure S13.** Funnel plot from genetically predicted PDE2A on Major depressive disorder.


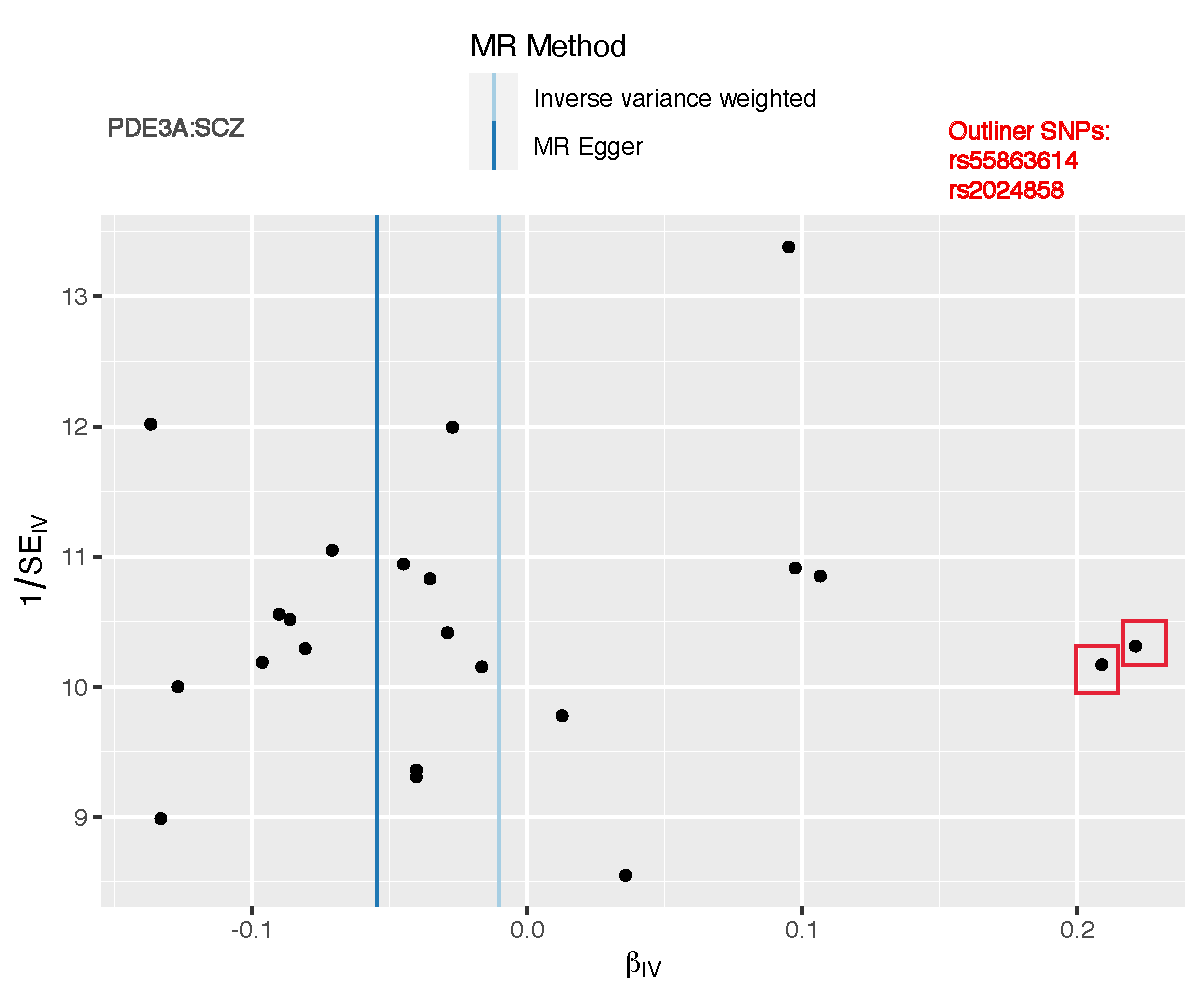


**Figure S14.** Funnel plot from genetically predicted PDE3A on Schizophrenia.


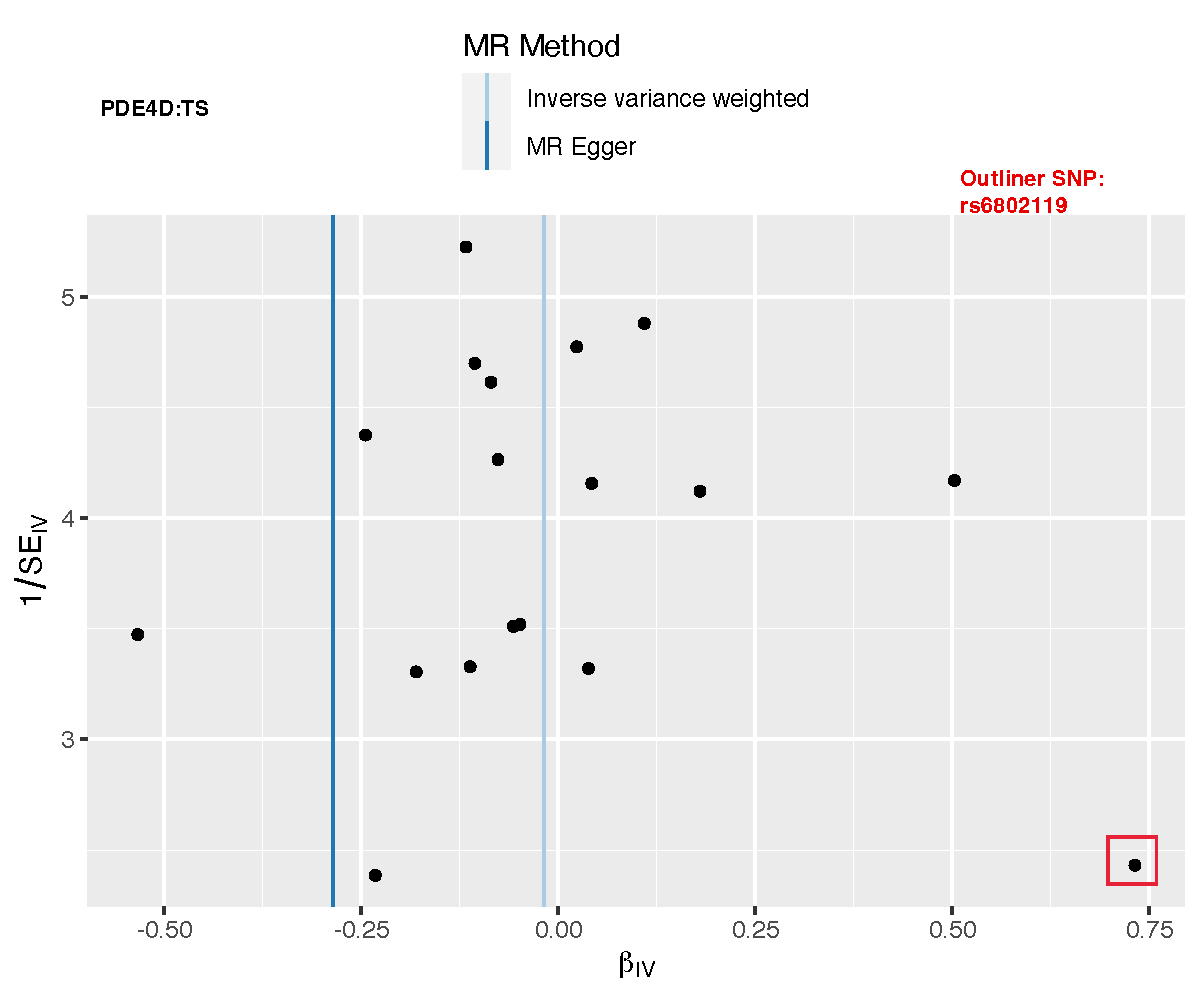


**Figure S15.** Funnel plot from genetically predicted PDE4D on Tourette syndrome.


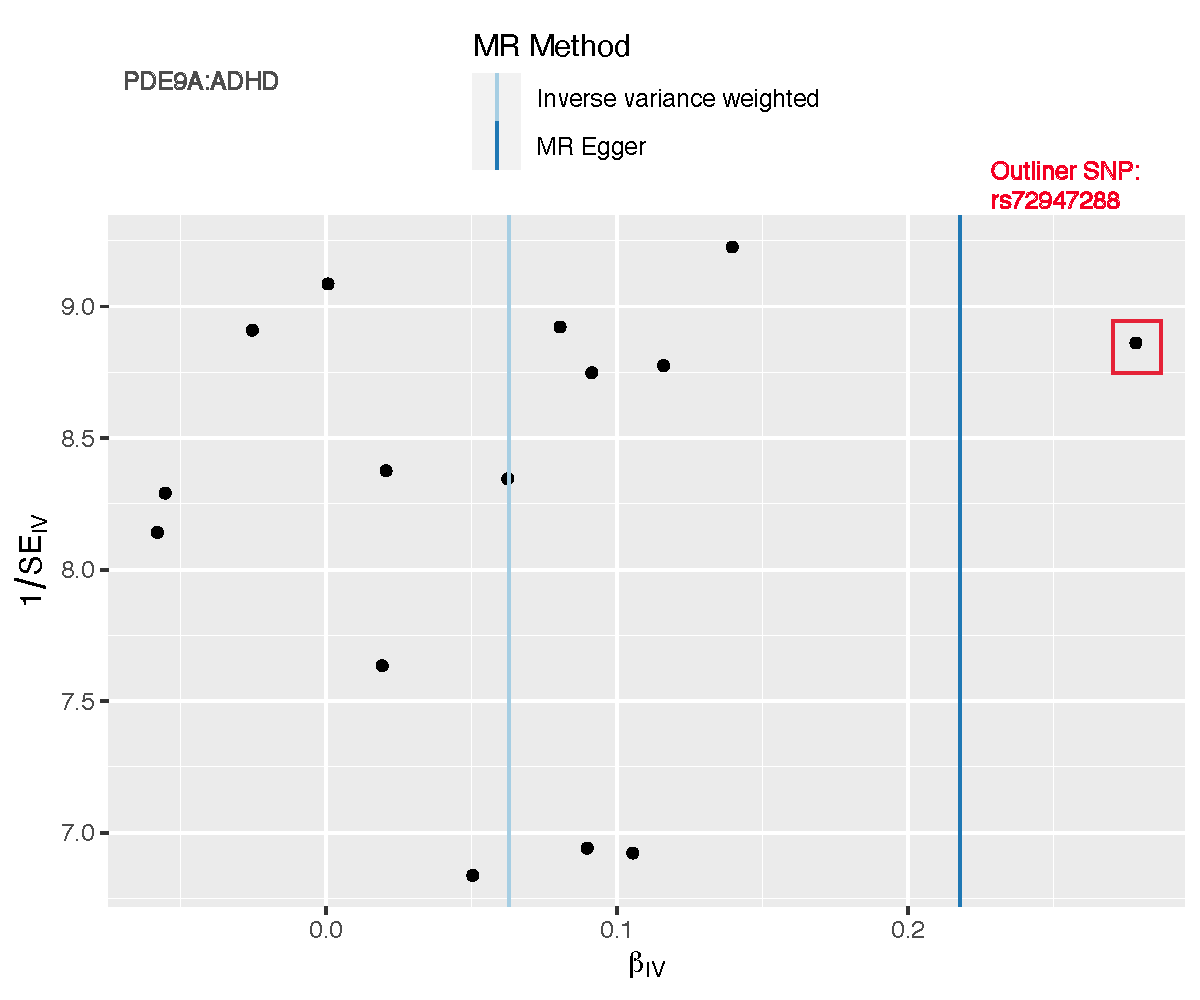


**Figure S16.** Funnel plot from genetically predicted PDE9A on Attention deficit hyperactivity disorder.


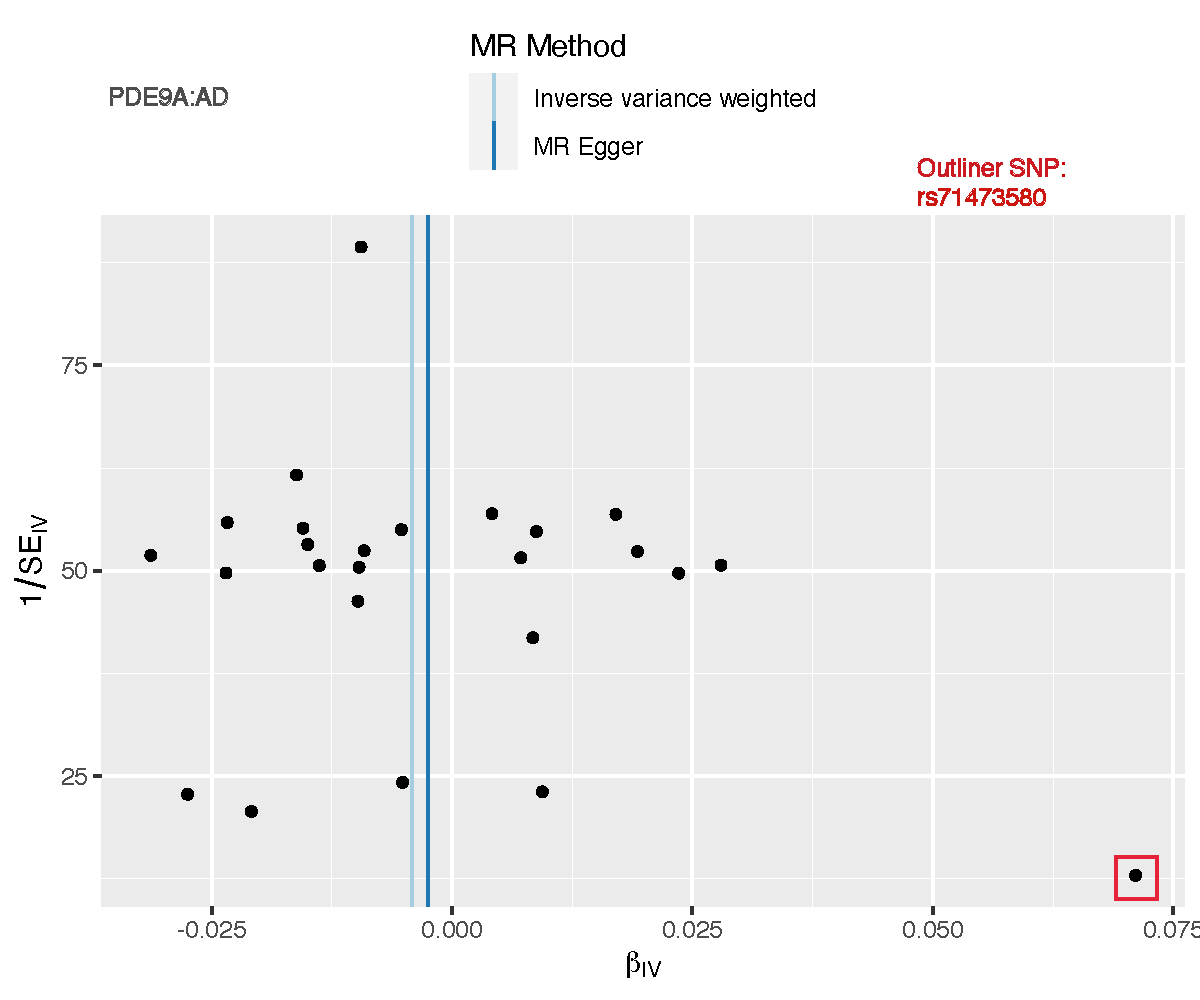


**Figure S17.** Funnel plot from genetically predicted PDE9A on Alzheimer's disease.


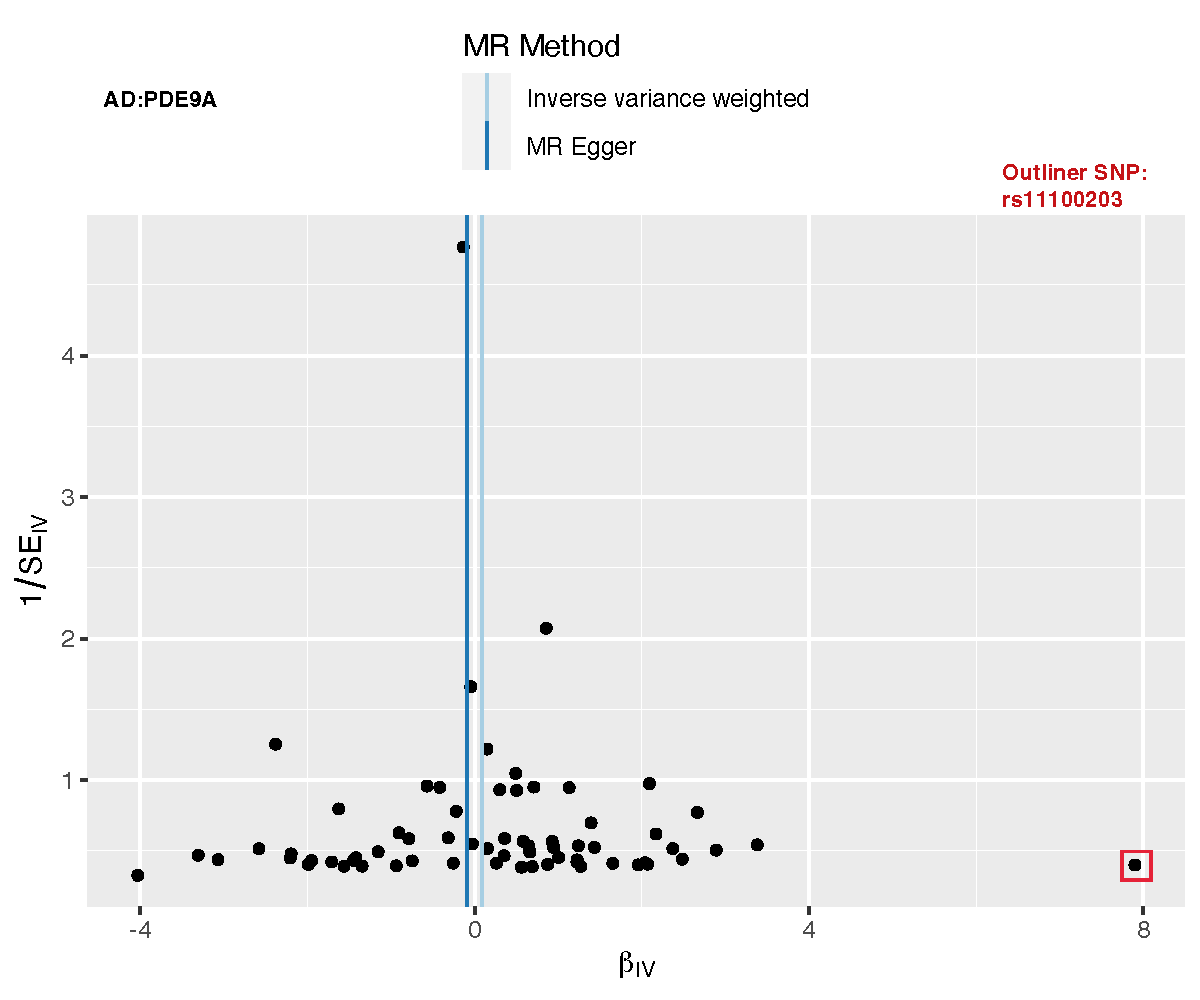


**Figure S18.** Funnel plot from genetically predicted Alzheimer's disease on PDE9A.


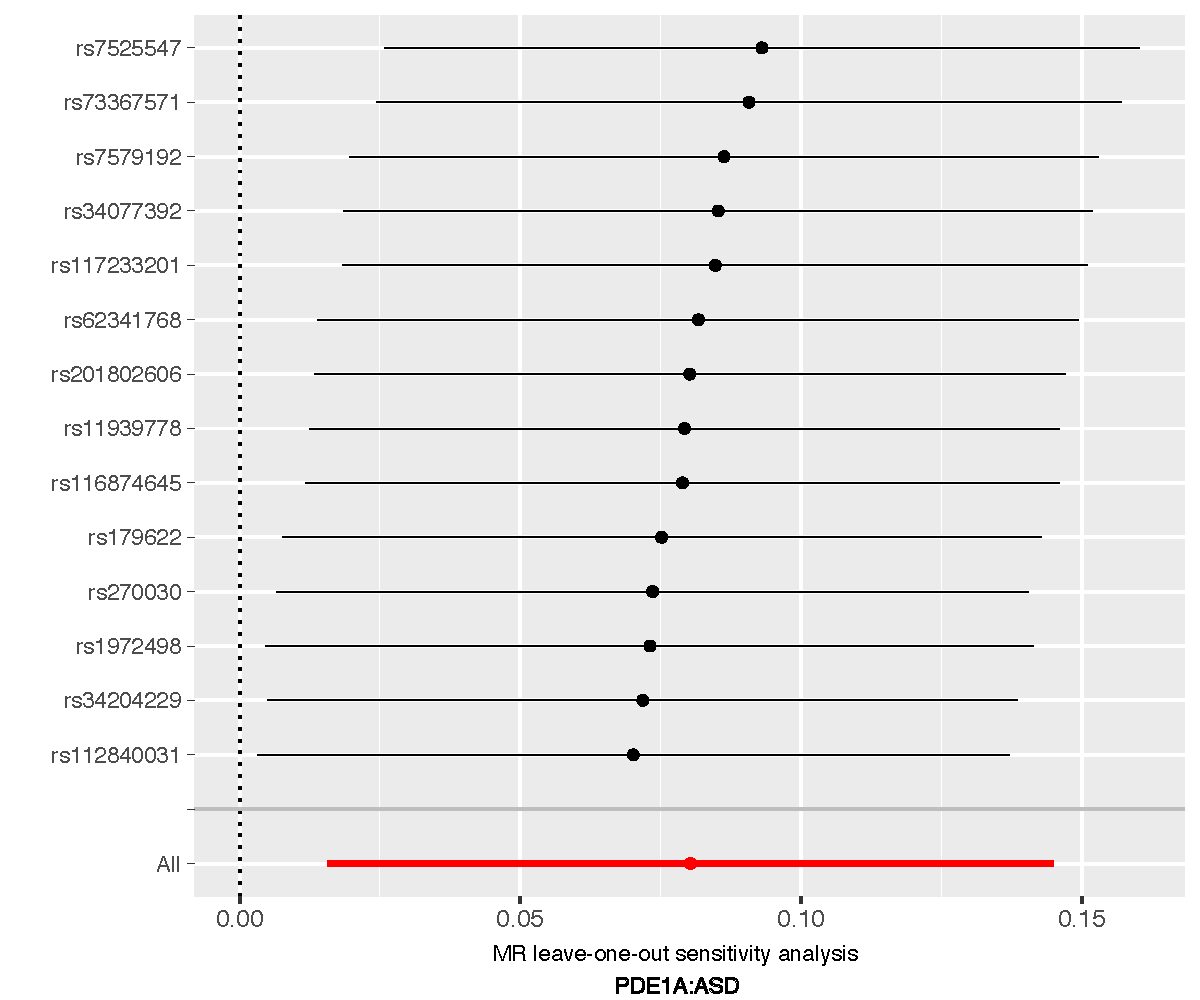


**Figure S19.** Leave-one-out analysis for PDE1A on Autism spectrum disorder.


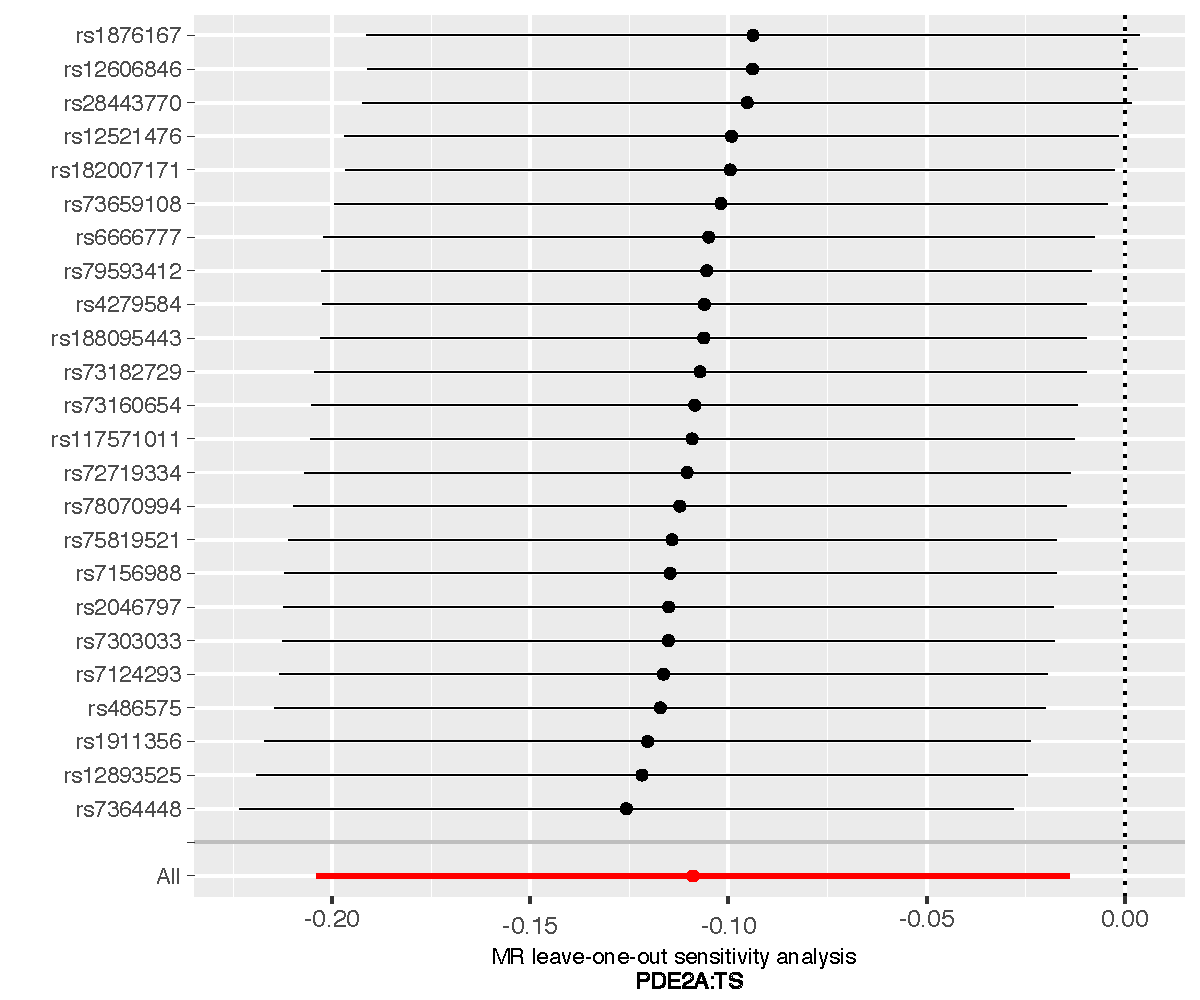


**Figure S20.** Leave-one-out analysis for PDE2A on Tourette syndrome.


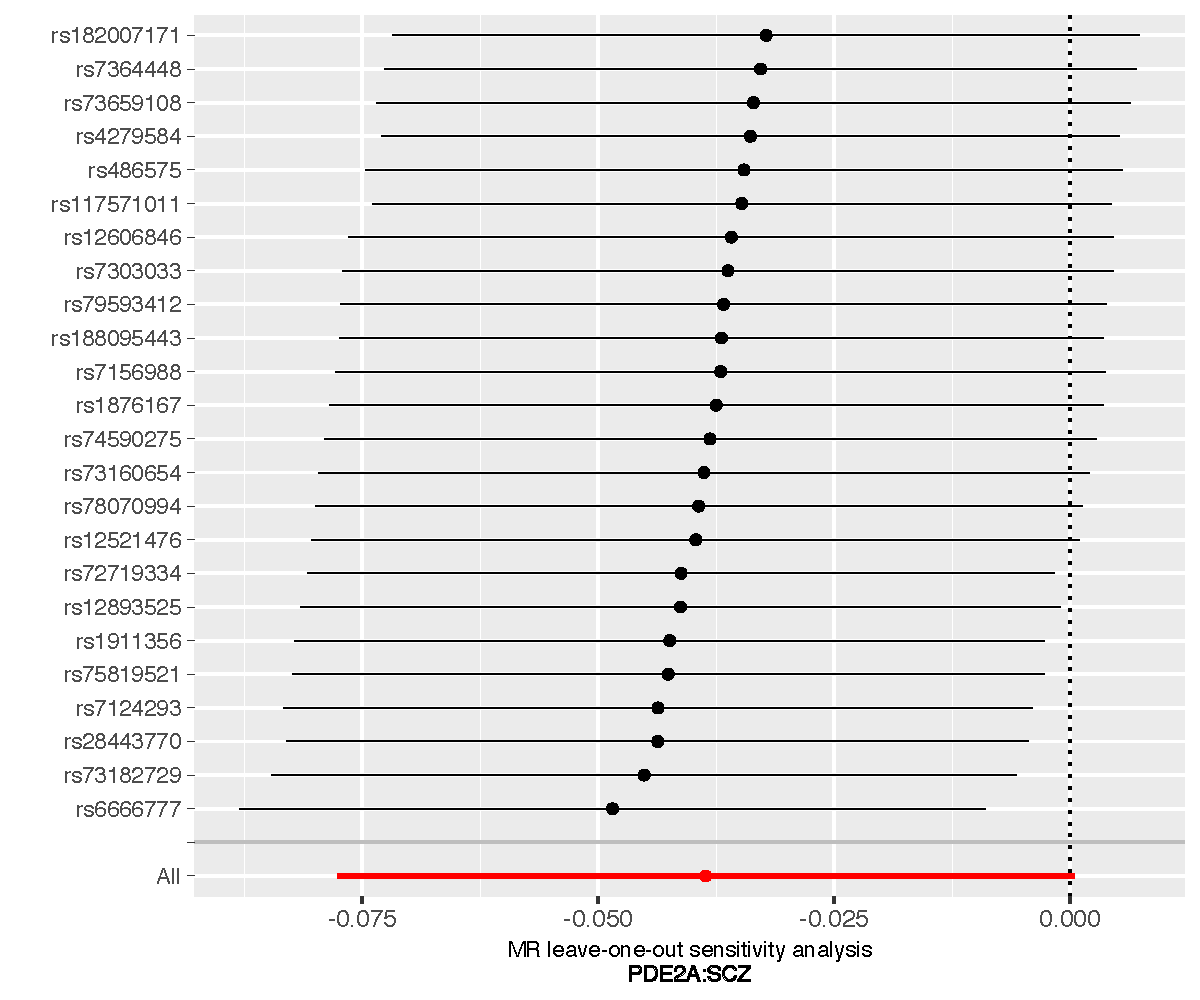


**Figure S21.** Leave-one-out analysis for PDE2A on Schizophrenia.

**
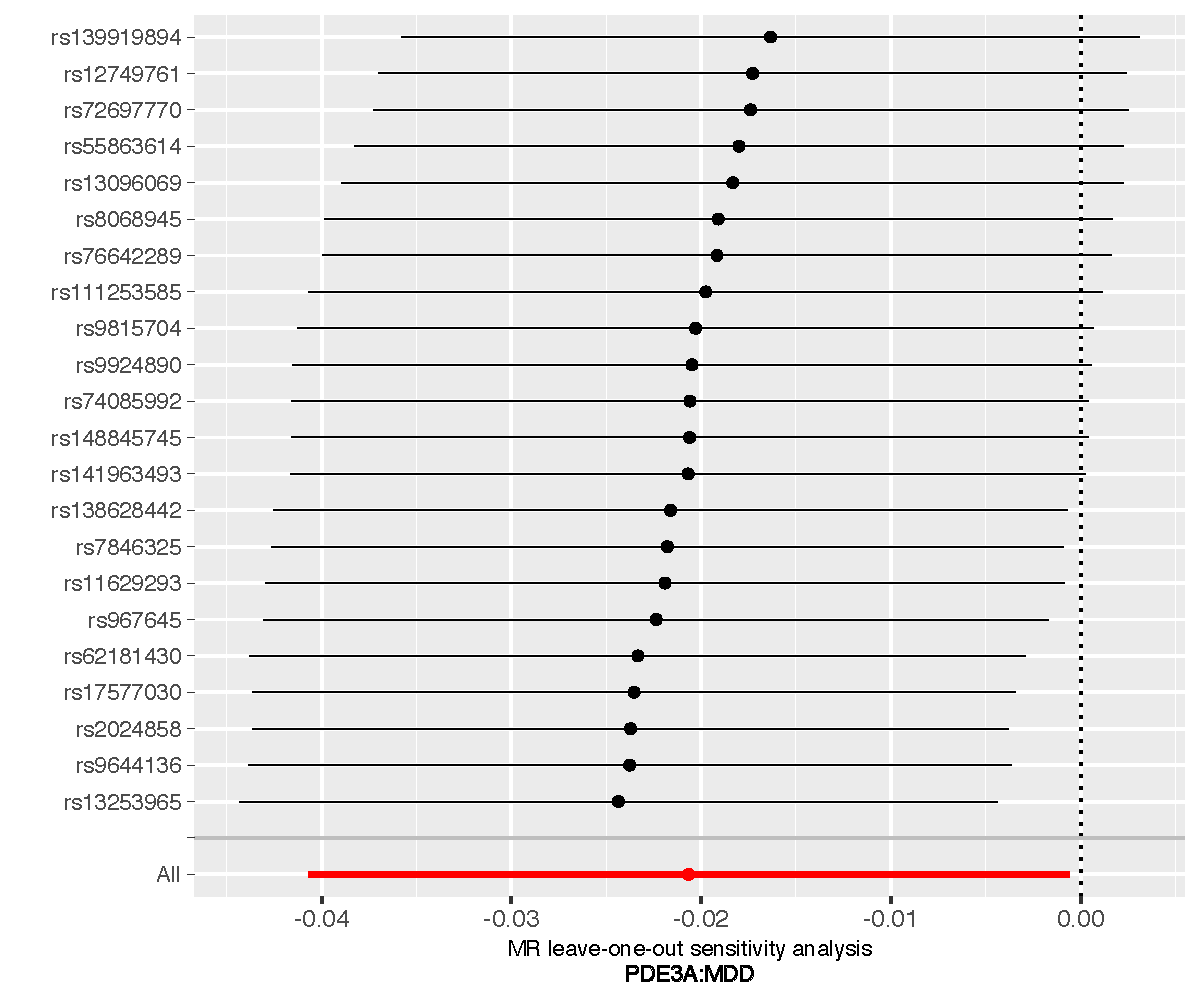
**

**Figure S22.** Leave-one-out analysis for PDE3A on Major depressive disorder.


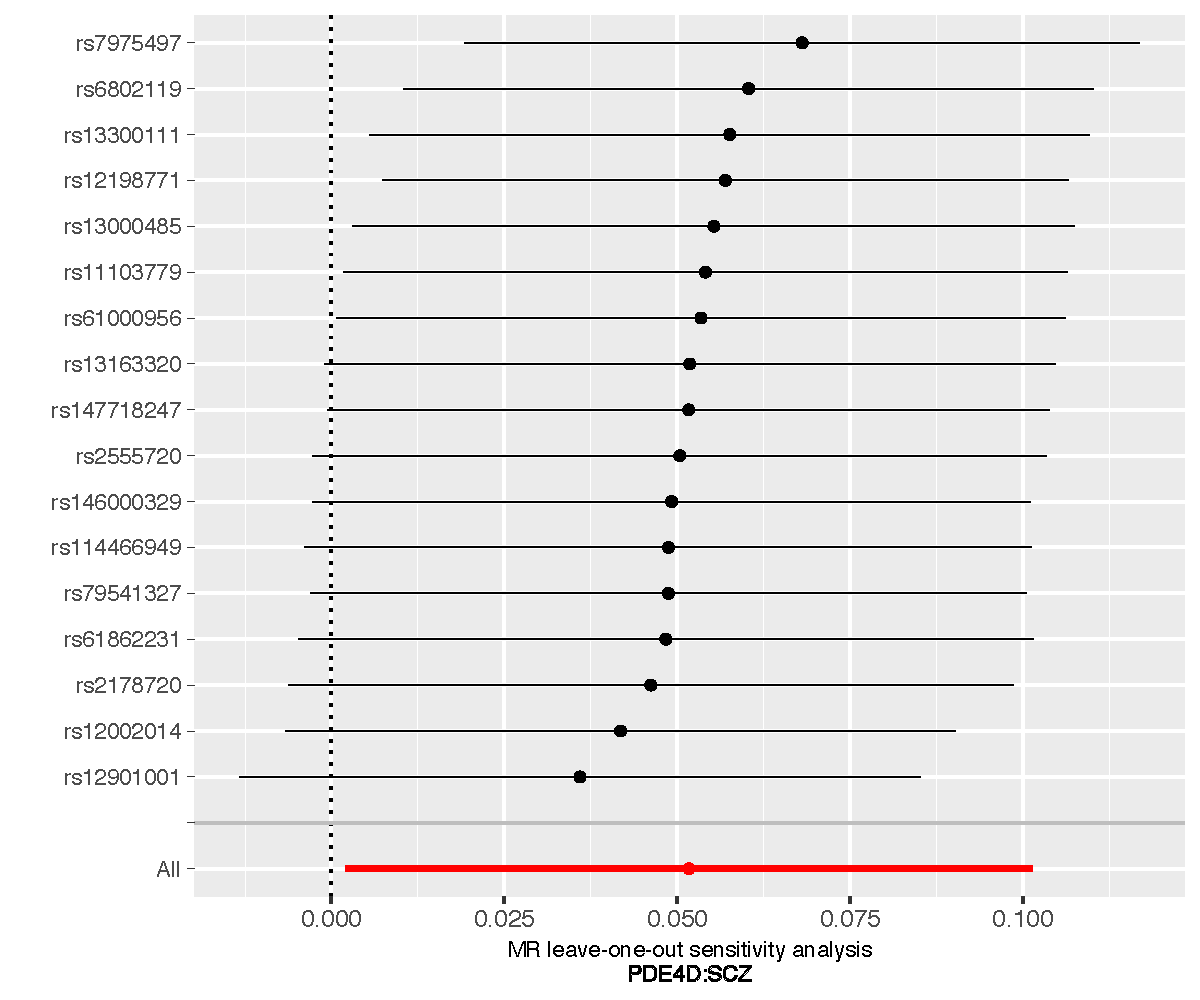


**Figure S23.** Leave-one-out analysis for PDE4D on Schizophrenia.

**
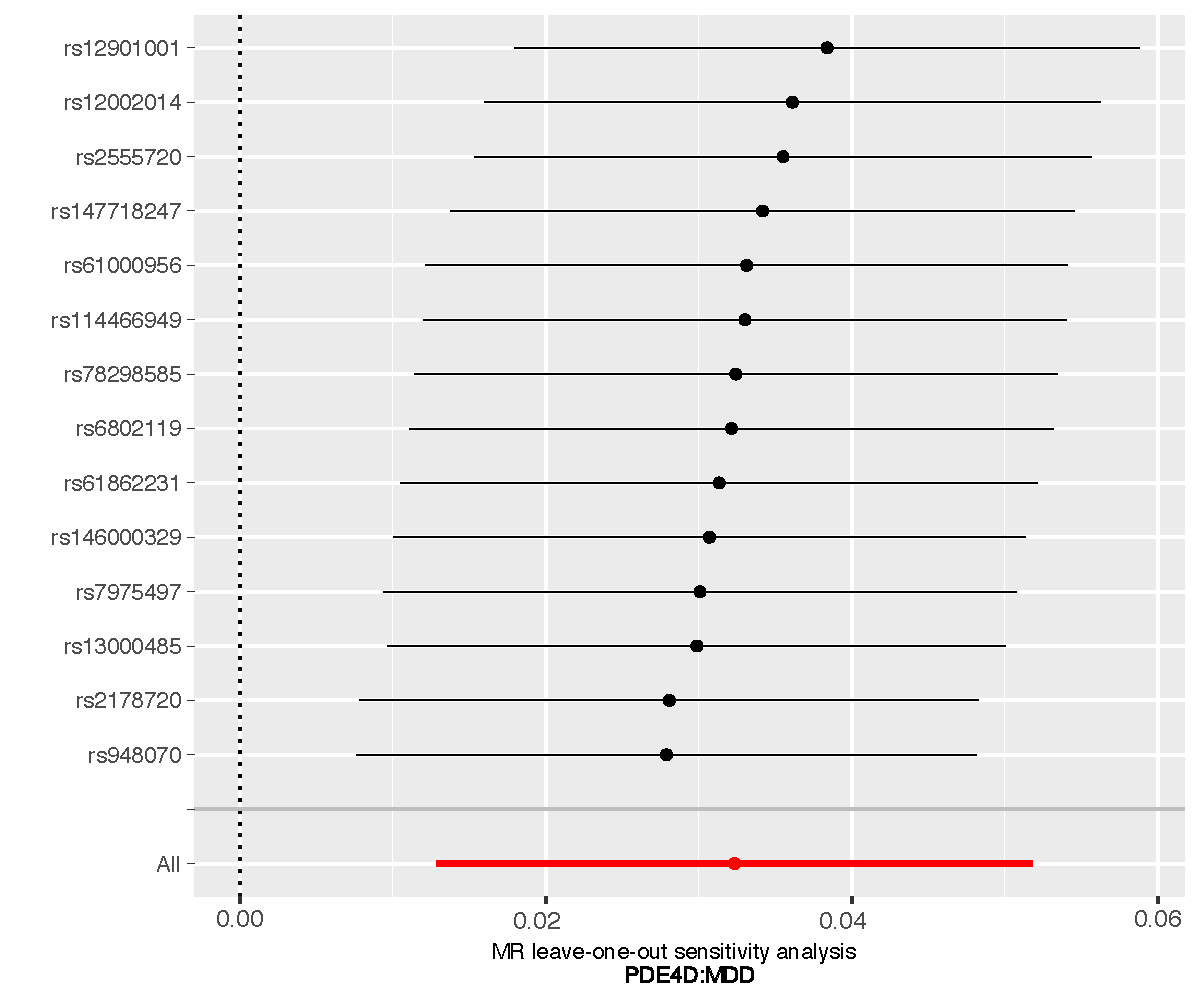
**

**Figure S24.** Leave-one-out analysis for PDE4D on Major depressive disorder.


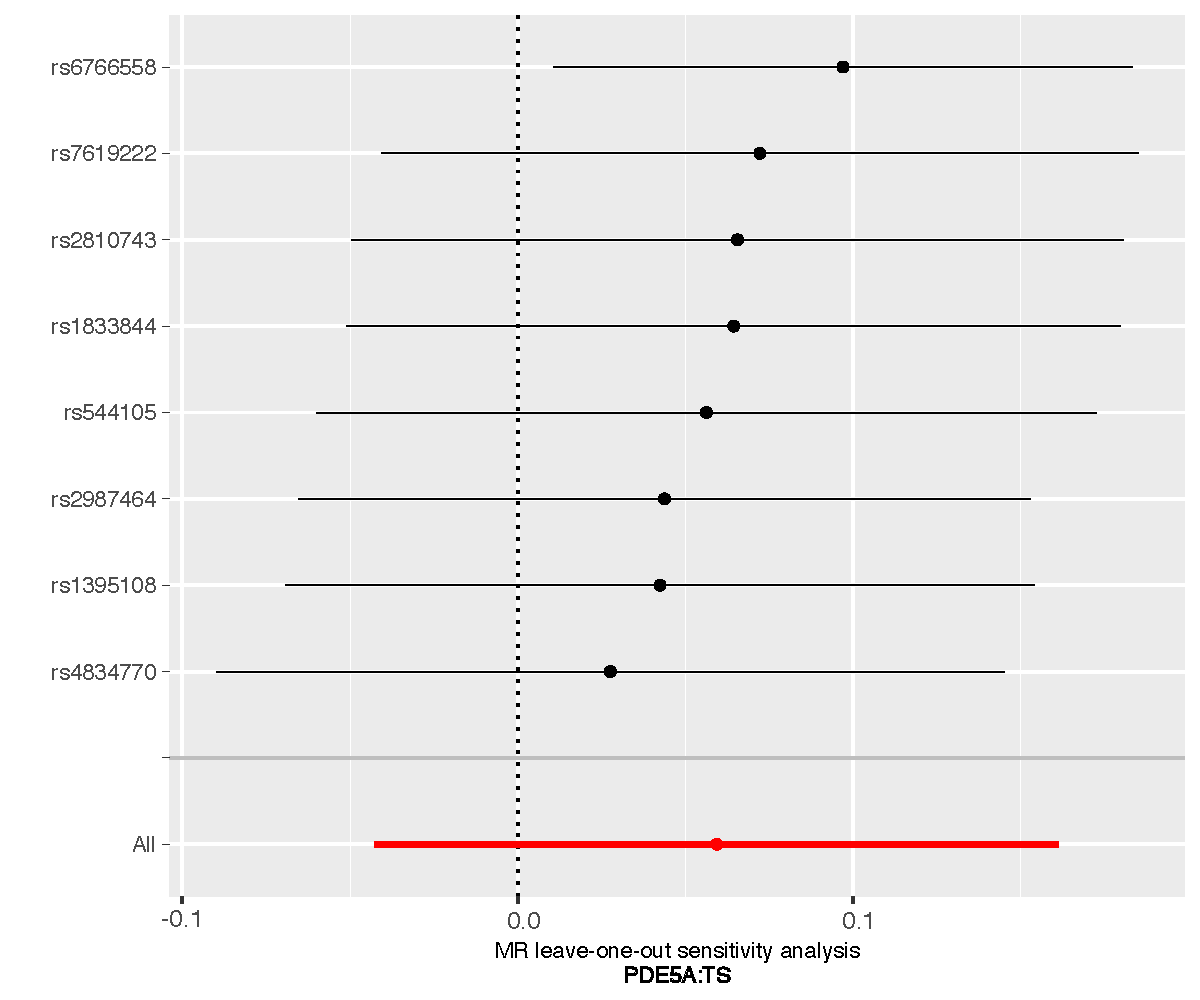


**Figure S25.** Leave-one-out analysis for PDE5A on Tourette syndrome.


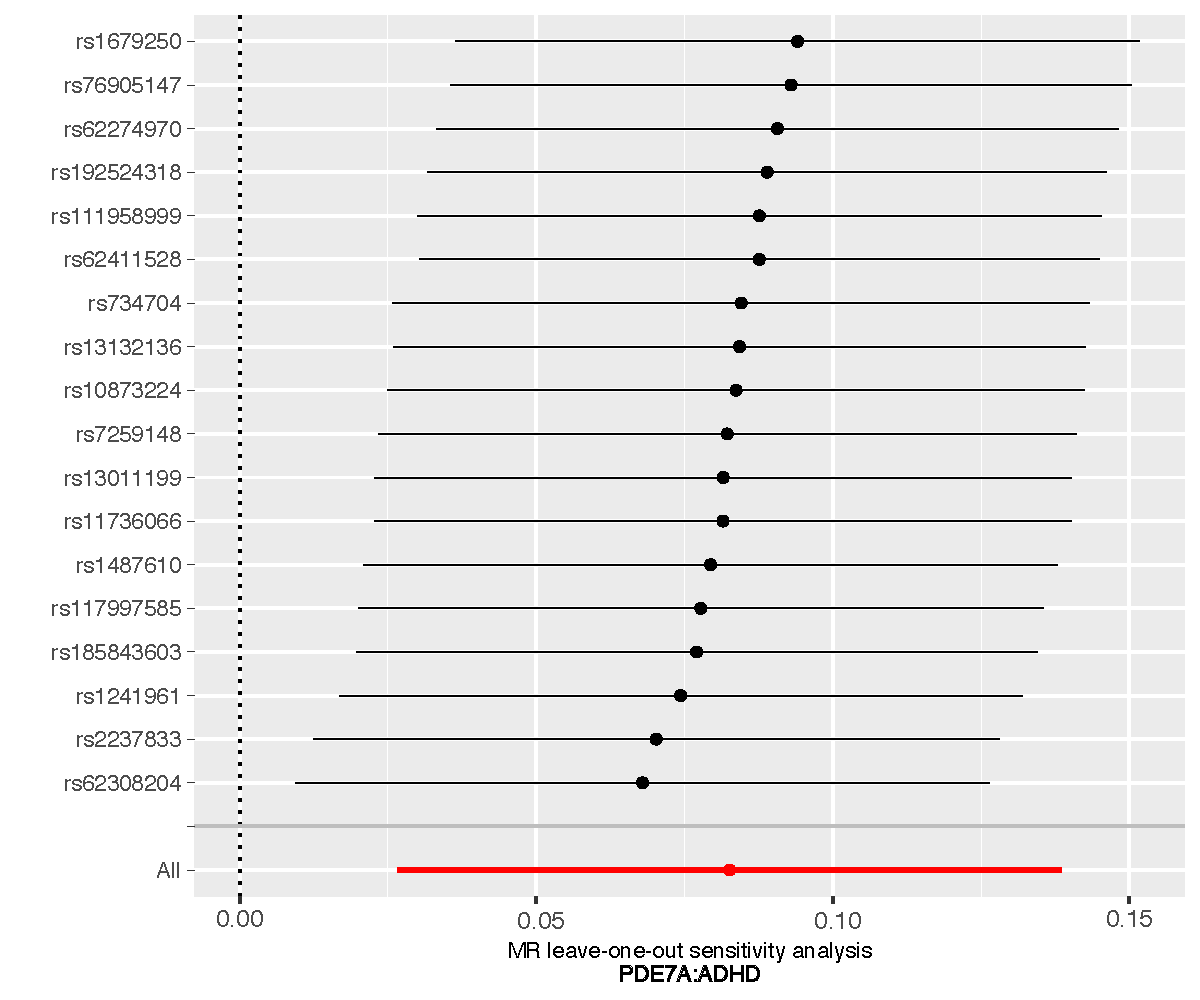


**Figure S26.** Leave-one-out analysis for PDE7A on Attention deficit hyperactivity disorder.


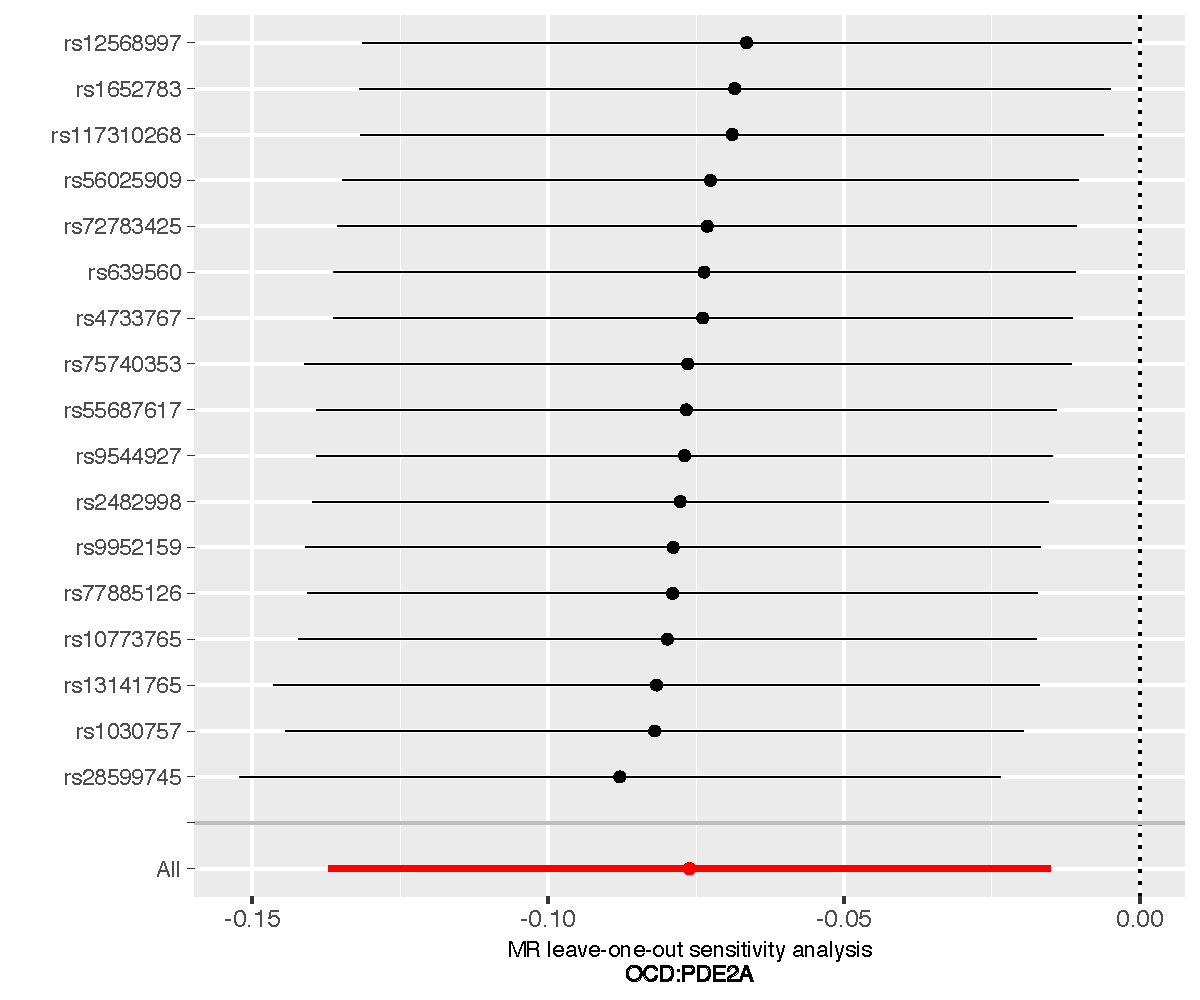


**Figure S27.** Leave-one-out analysis for Obsessive-compulsive disorder on PDE2A.


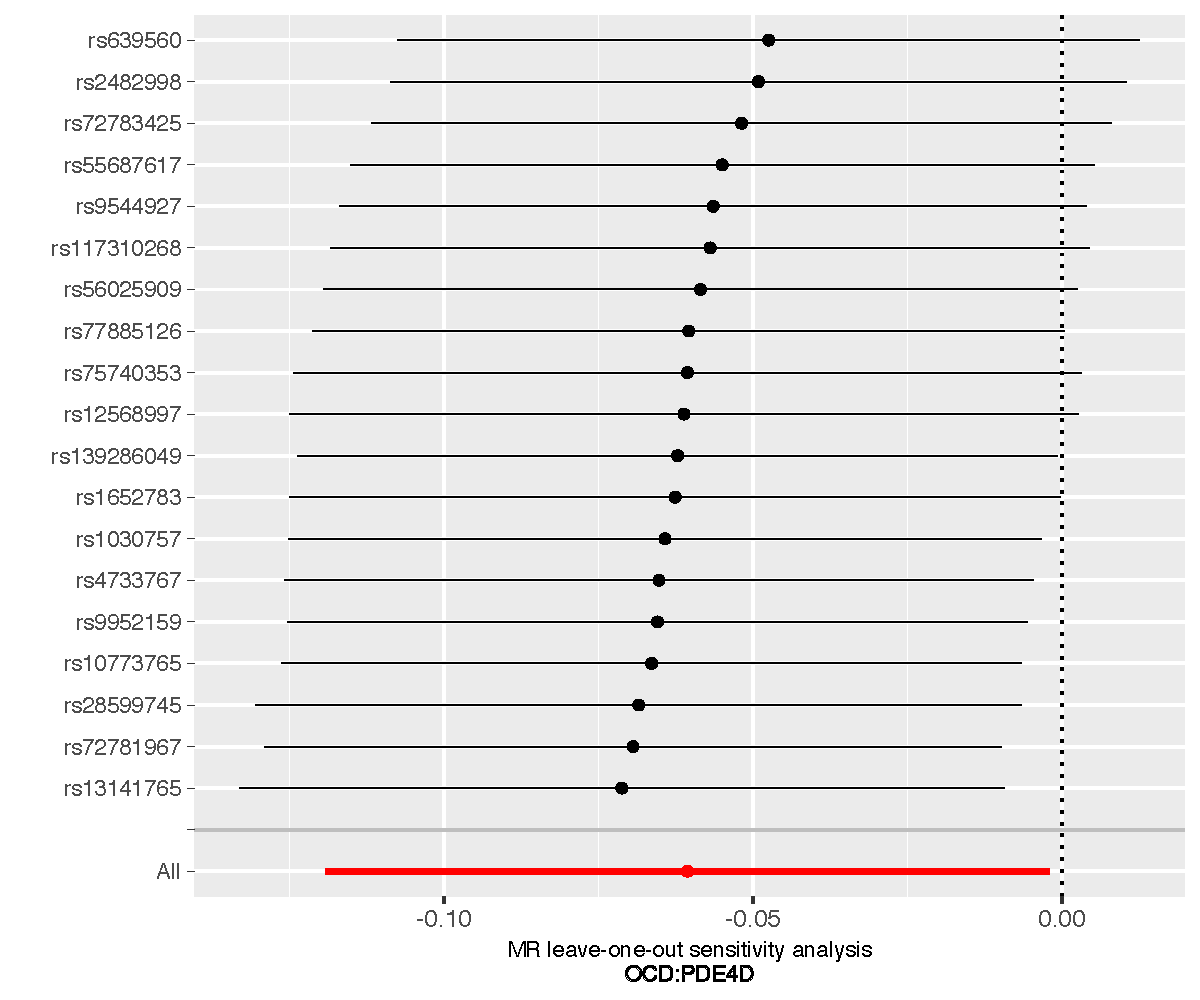


**Figure S28.** Leave-one-out analysis for Obsessive-compulsive disorder on PDE4D.


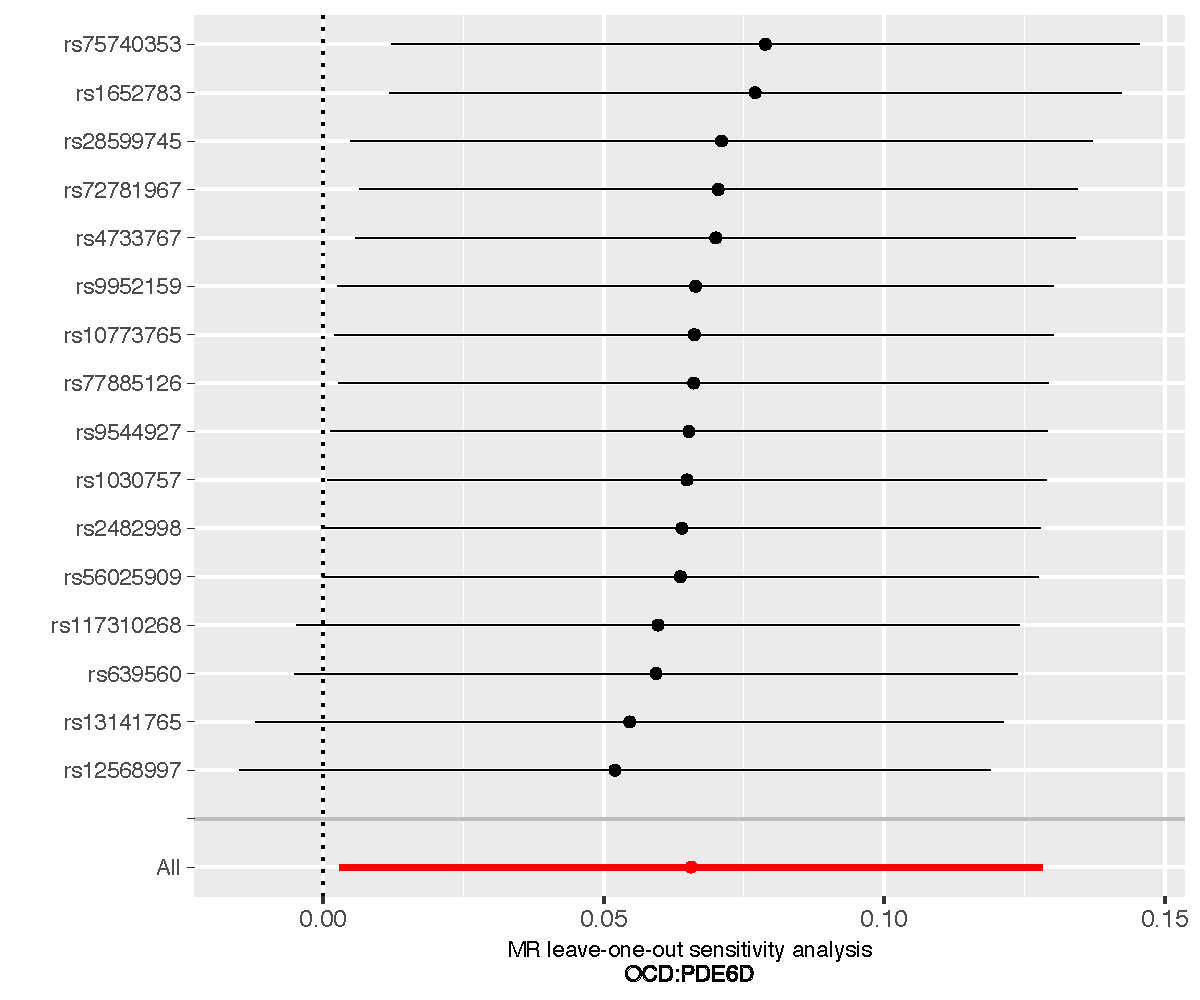


**Figure S29.** Leave-one-out analysis for Obsessive-compulsive disorder on PDE6D.


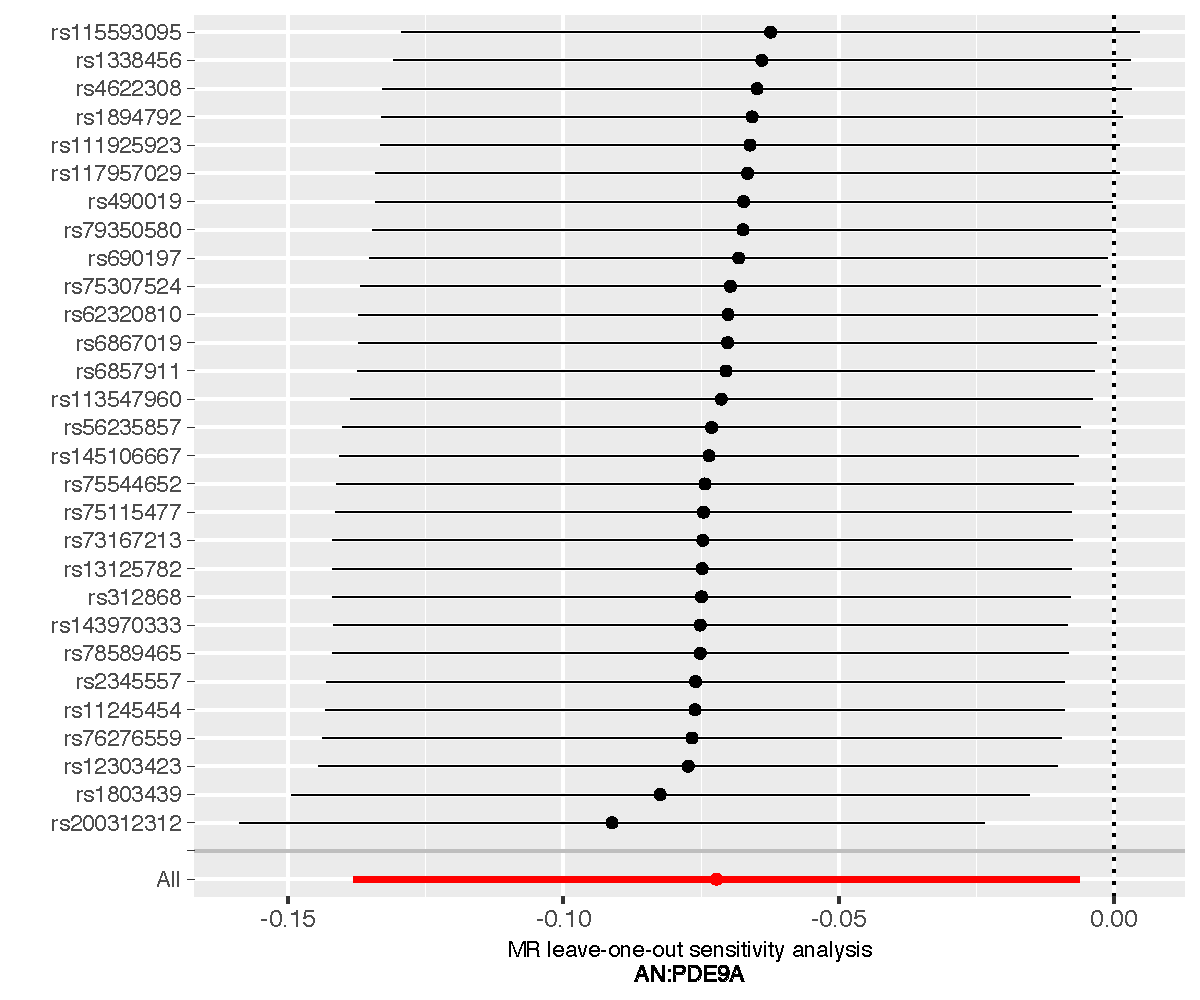


**Figure S30.** Leave-one-out analysis for Anorexia nervosa on PDE9A.


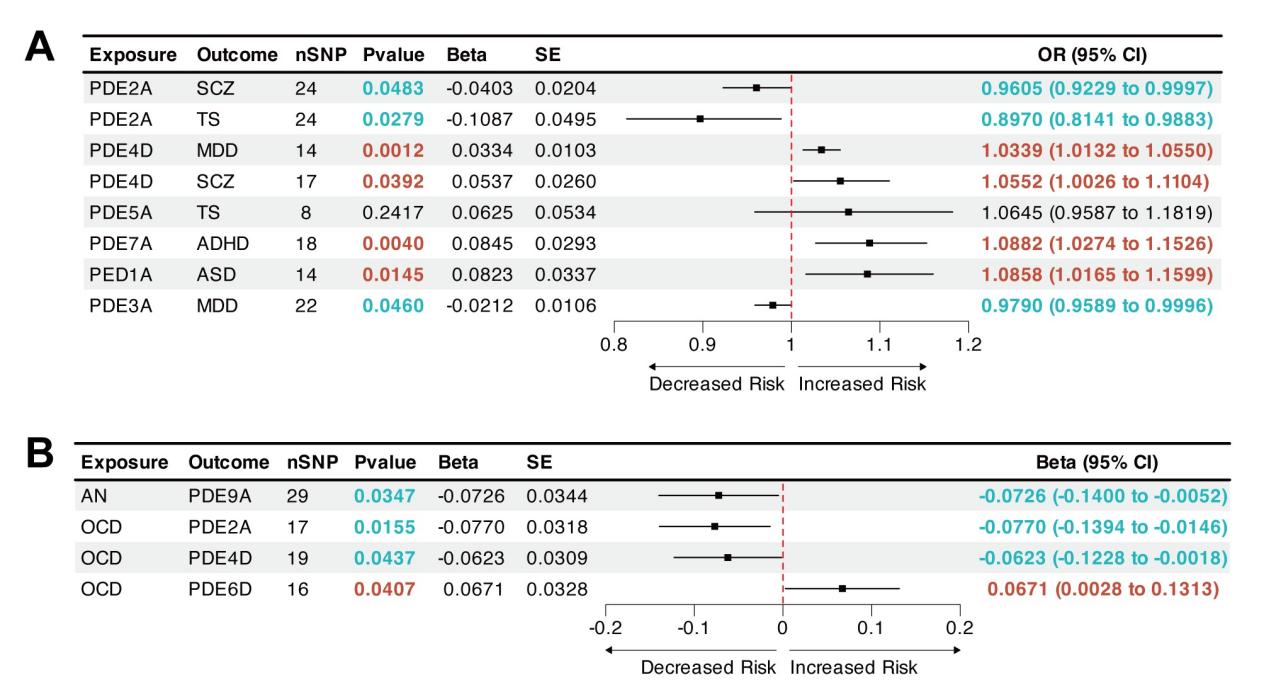


**Figure S31.** The forest plot shows the significant causalities by maximum likelihood (ML) methods.

Associations between genetically predicted phosphodiesterases (PDEs) and the risk of psychiatric disorders (A). Associations between genetically predicted psychiatric disorders and PDEs (B). No, number; SNP, single nucleotide polymorphism; Beta, genetic effect size from the exposure GWAS data; SE, standard error of effect size; OR, odds ratio.
